# Engineering a triple-functional magnetic gel driving mutually-synergistic mild hyperthermia-starvation therapy for osteosarcoma treatment and augmented bone regeneration

**DOI:** 10.1186/s12951-023-01955-7

**Published:** 2023-06-26

**Authors:** Kexiao Yu, Hang Zhou, Yamei Xu, Youde Cao, Yuanyi Zheng, Bing Liang

**Affiliations:** 1 Department of Orthopedics, Chongqing Traditional Chinese Medicine Hospital, No. 6 Panxi Seventh Branch Road, Jiangbei District, Chongqing, 400021 P. R. China; 2 Department of Orthopedics, Second Affiliated Hospital of Chongqing Medical University, 76 Linjiang Road, Yuzhong Distinct, Chongqing, 400010 P. R. China; 3 Department of Pathology, College of Basic Medicine, Molecular Medicine Diagnostic and Testing Center, Chongqing Medical University, 1 Yixueyuan Road, Yuzhong Distinct, Chongqing, 400016 P.R. China; 4 Department of Ultrasound in Medicine, Shanghai Institute of Ultrasound in Medicine, Shanghai Jiao Tong University Affiliated Sixth People’s Hospital, 600 Yishan Road, Xuhui Distinct, Shanghai, 200233 P. R. China; 5 State Key Laboratory of Ultrasound in Medicine and Engineering, Institute of Ultrasound Imaging, Chongqing Medical University, Chongqing, 400010 People’s Republic of China

**Keywords:** Triple-functional magnetic hydrogels, Mild hyperthermia therapy, Starvation therapy, Osteosarcoma, Bone regeneration

## Abstract

Malignant bone tumors result in high rates of disability and death and are difficult to treat in terms of killing tumors and repairing bone defects. Compared with other hyperthermia strategies, magnetic hyperthermia has become an effective therapy for treating malignant bone tumors due to its lack of depth limitations. However, tumor cells express heat shock protein (HSP) to resist hyperthermia, which reduces its curative effect. Competitive ATP consumption can reduce HSP production; fortunately, the basic principle of starvation therapy by glucose oxidase (GOx) is consuming glucose to control ATP production, thereby restricting HSP generation. We developed a triple-functional magnetic gel (Fe_3_O_4_/GOx/MgCO_3_@PLGA) as a magnetic bone repair hydrogels (MBRs) with liquid‒solid phase transition capability to drive magneto-thermal effects to simultaneously trigger GOx release and inhibit ATP production, reducing HSP expression and thereby achieving synergistic therapy for osteosarcoma treatment. Moreover, magnetic hyperthermia improves the effect of starvation therapy on the hypoxic microenvironment and achieves a reciprocal strengthening therapeutic effect. We further demonstrated that in situ MBRs injection effectively suppressed tumor growth in 143B osteosarcoma tumor-bearing mice and an in-situ bone tumor model in the rabbit tibial plateau. More importantly, our study also showed that liquid MBRs could effectively match bone defects and accelerate their reconstruction via magnesium ion release and enhanced osteogenic differentiation to augment the regeneration of bone defects caused by bone tumors, which generates fresh insight into malignant bone tumor treatment and the acceleration of bone defect repair.

**Supplementary Information:**

The online version contains supplementary material available at 10.1186/s12951-023-01955-7.

## Introduction

Malignant bone tumors, especially osteosarcoma (OS), are reinforced by the special anatomical structure of lesion sites and their corresponding physiological barriers, which decrease the effects of traditional radiotherapy and chemotherapy [1, 2]. Compared with other solid tumors, OS causes the terrible erosion of bone tissues, which can lead to severe bone defects and even pathological fractures [3]. More disturbingly, overtreating the tumors with open surgery results in the destruction of the skeletal stress structure, limiting patients’ ability to walking. Therefore, simultaneously treating OS and repairing bone defects to reconstruct the stress conduction of bone is difficult in clinical treatment, especially in minimally invasive approaches. To facilitate the development of desirable biomaterials for integrative therapy and repair, various techniques and methods have been proposed for their synthesis, such as casting, 3D printing and selective laser sintering [4]. These bone tissue engineering materials have good support performance; however, due to their poor performance in filling irregular defects caused by pathological fractures, lack of degradability and insufficient drug release properties, their application in treating OS has been limited. Hence, the synthesis of injectable multifunctional biomaterials with superior shape adaptability, biodegradability and good drug release capability may be significant for anti-OS therapy. In our previous studies, we prepared an injectable phase-transform poly(lactide-co-glycolide) (PLGA)/1-methyl-2-pyrrolidinone (NMP) solution as a PLGA hydrogel, which was used for drug release by intratumor injection after liquid‒solid transformation [5, 6]. In particular, PLGA gels, as porous biomimetic scaffold implants with good biosafety and biodegradability, show great potential as loaded functional nanoparticles for treating bone tumors via a minimally invasive procedure.

Magnetic hyperthermia therapy (MHT), as a noninvasive treatment modality, has been widely used in clinical antitumor applications due to its superb benefits, namely, the lack of a depth penetration limit, the long-range control, and various performance therapeutic modes [7–9]. It can be used as the main therapeutic method or as an adjuvant therapy to enhance the effect of other therapies, such as chemotherapy, radiotherapy and supplemental therapy, after surgery. Compared with photothermal therapy, MHT, with its ability to access deep bone structures, is ideally suited to achieving good therapeutic effects for bone tumors [10]. Because tumor cells have a temperature sensitivity greater than that of normal cells, this specialized therapy has been utilized in local tumor thermal therapy to prevent surrounding normal tissue damage. However, the intrinsic thermoresistance of tumor cells, arising from the upregulation of heat shock proteins (HSPs) during MHT, is inextricably problematic for improving the curative effect [11, 12]. Our previous studies and some reports showing that residual tumors, recurrence and metastasis are accompanied by generation of large amounts of HSPs strongly demonstrated that this phenomenon is key for boosting therapeutic outcomes [13]. Many valuable strategies have been proposed to restrict HSP generation, for example, by interlinking HSP inhibitors [14–16], such as siRNA. However, the targeted delivery of small-molecule inhibitors is difficult due to the lack of serum stability, low intracellular uptake rate, and escape by body circulation. Hence, there is an urgent need to engineer a biomaterial to reduce the tumor heat resistance elicited by HSPs and enhance the efficiency of MHT.

Based on the Warburg effect, the metabolism and proliferation e depend on much supplied glucose [17, 18]. HSP overexpression is correlated with the energy supply of adenosine triphosphate (ATP) by glucose consumption, which presents a possible strategy for downregulating HSP expression [19]. Several studies have reached a consensus that glucose oxidase (GOx), a starvation therapy mediator, can transform intratumoral glucose and oxygen into gluconic acid and hydrogen peroxide (H_2_O_2_) [20–22]. It could competitively inhibit the tricarboxylic acid cycle to reduce ATP and thereby suppress HSP generation, thus augmenting the MHT effect. However, the hypoxic intratumoral microenvironment and continuous accumulation of acid products limit the reaction process, presenting obstacles to the use of this antitumor therapy [23, 24]. Hence, if the GOx-enzymatic reaction could be promoted by decomposing a specific compound in the tumor environment, such as H_2_O_2_, this would not only greatly inhibit HSP generation but also accelerate glucose expenditure to obtain the mutual synergistic therapeutic efficacy of osteosarcoma treatment.

**Scheme 1:**
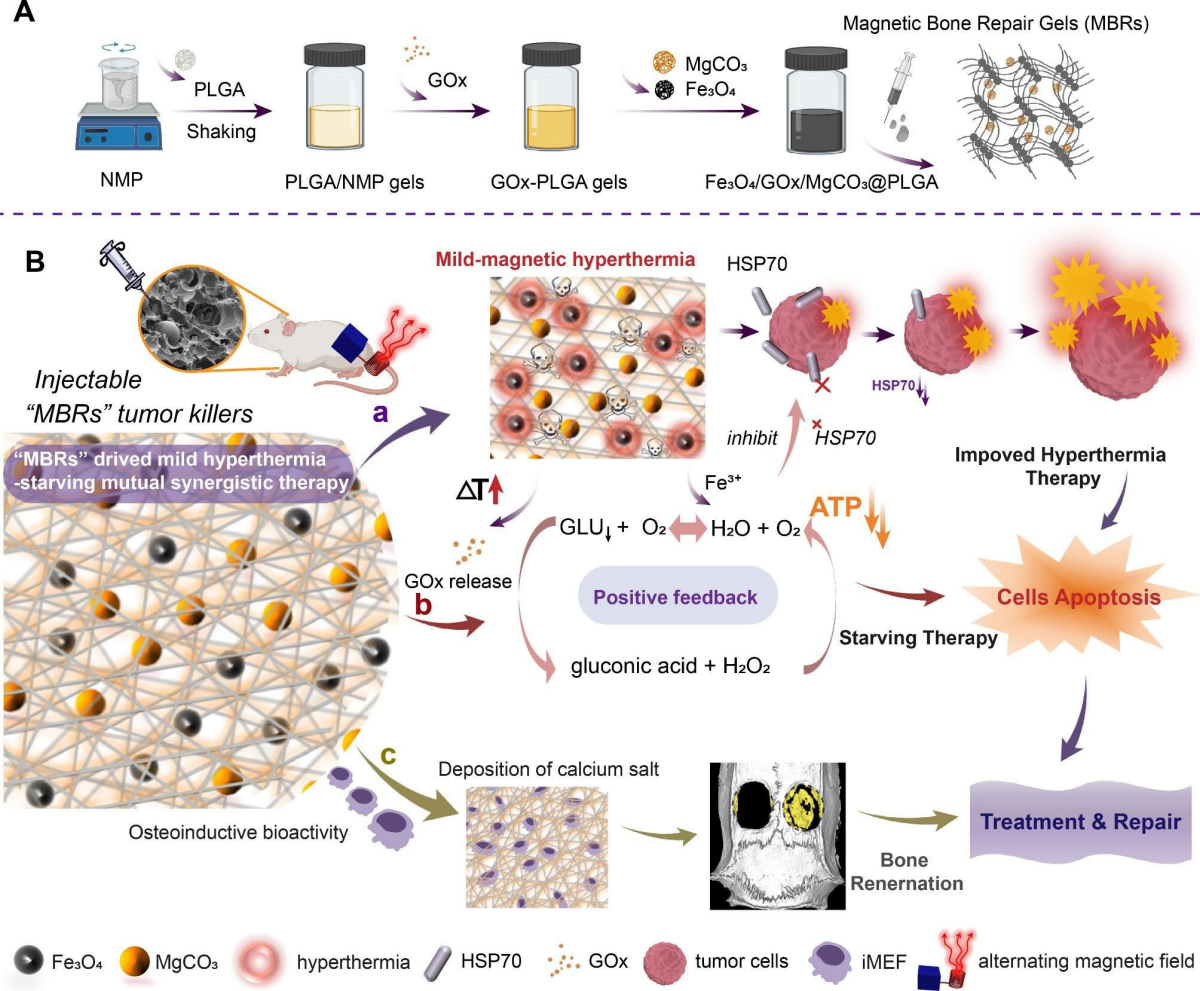
Schematic illustration of the mechanism by which injectable multifunctional magnetic bone repair hydrogels (MBRs), such as Fe_3_O_4_/GOx/MgCO_3_@PLGA, drive mild hyperthermia-starving mutual synergistic therapy for bone tumor treatment and accelerate bone defect repair. (**A**) Synthesis of PLGA gels and the encapsulation of glucose oxidase and Fe_3_O_4_/MgCO_3_ nanoparticles. (**B**) Mild hyperthermia-triggered GOx release to induce starvation-magnetic synergistic therapy in 143B bone tumors. The enzymatic activity of GOx was reserved under mild thermal conditions and accelerated the enzyme-promoting reaction, and HSP70 was inhibited by simultaneously decreasing ATP for enhanced magnetic hyperthermia therapy. The irregular bone defect was repaired by the porous structure of the MBRs, enhanced osteogenic differentiation and calcium salt deposition to achieve a treatment and repair two-in-one strategy

In all of the aforementioned approaches, maintaining the bioactivity and efficiency of GOx under thermal triggering plays a crucial role. In addition, thoroughly removing the tumor and rebuilding the bone structure are still dilemmas. In this work, we designed injectable phase-transform PLGA gels loaded with Fe_3_O_4_ particles and encapsulated GOx to design a mutual synergistic therapy that simultaneously drives magnetic hyperthermia and starvation therapy (Scheme 1 A). With the good fluidity of the PLGA gels, the triple-functional magnetic gels were easily injected into tumors and filled irregular pathological bone defects. Under alternating magnetic field (AMF) exposure, the Fe_3_O_4_ nanoparticles triggered magnetic thermal therapy, making the solid form gels more porous to drive GOx release into the tumor. To prevent damage to the surrounding normal tissue, we used an “on-off” multicycle method to control the treatment temperature at approximately 40–45 °C and prevent GOx deactivation at high temperatures. Additionally, Fe^3+^ and an appropriate temperature were used as catalysts to facilitate H_2_O_2_ decomposition into H_2_O and O_2_, achieving a positive reaction feedback (Scheme 1B). More importantly, this enzymatic reaction consumes a large amount of glucose to inhibit ATP synthesis, consequently leading to HSP downregulation to reduce the thermal resistance of osteosarcoma cells, achieving mutual synergy between mild hyperthermia and starvation therapy. Simultaneously, after treating osteosarcoma, the gels filled the bone defects and facilitated repair via a MgCO_3_-loaded compound as Fe_3_O_4_/GOx/MgCO_3_@PLGA gels, denoted magnetic bone repair hydrogels (MBRs). The porous structure is beneficial to osteoblast creep and adhesion. Moreover, appropriate and sustainable Mg^2+^ release endowed the MBRs with osteoinductive bioactivity, increasing osteoblastic differentiation in vitro and bone regeneration in vivo. Due to the special anatomical structure of bone tumors and the current susceptibility to recurrence after surgical treatment, we constructed an in situ residual bone tumor model in the rabbit tibial plateau, verifying that MBRs can treat residual tumors and inhibit tumor recurrence. Specifically, the good biosafety of MBRs was verified, enabling further promotion of this new therapeutic strategy. In summary, MBR gels with triple-functional performance drive mutual synergy between mild hyperthermia and starvation therapy for osteosarcoma treatment and augment bone regeneration via a minimally invasive procedure, holding great promise for malignant bone tumor treatment and the repair of pathological bone defects.

## Results and discussion

### Structure and composition characteristics of PLGA and MBR gels

The preparation method of implants of minimally invasive carriers should be efficient and convenient. To construct the bioactive MBRs, PLGA/NMP solution (PLGA gels) was first obtained, as shown in Scheme 1 A. The liquid PLGA gels were synthesized by shaking PLGA particles with NMP solution. PLGA, a compound approved by the U.S. Food and Drug Administration (FDA) for clinical use, has been used as a sustained-release drug carrier, artificial catheter, and tissue engineering scaffold material in several treatment fields, which suggests its further potential in tumor therapeutics [25, 26]. The synthetic PLGA gels with a good liquid fluidity could also be equally loaded with multiple functional particles, such as Fe_3_O_4_, GOx and MgCO_3_. After the dispersion of small particles of Fe_3_O_4_, GOx and MgCO_3_ (Figure S1A-C) into the composite PLGA gels, the MBRs were easily obtained as black gels (Figure S2). Notably, solid PLGA has a porous structure (Figure S1D), which suggests the ability to release hydrosoluble GOx into its surroundings. As shown in Figure S3, MBRs remained a homogeneous mixture even after 8 h, implying that the Fe_3_O_4_, GOx and MgCO_3_ particles could achieve stable distribution in the PLGA gel. Importantly, the excellent stability of MBRs endows operators with sufficient time for hydrogel implantation.

**Fig. 1:**
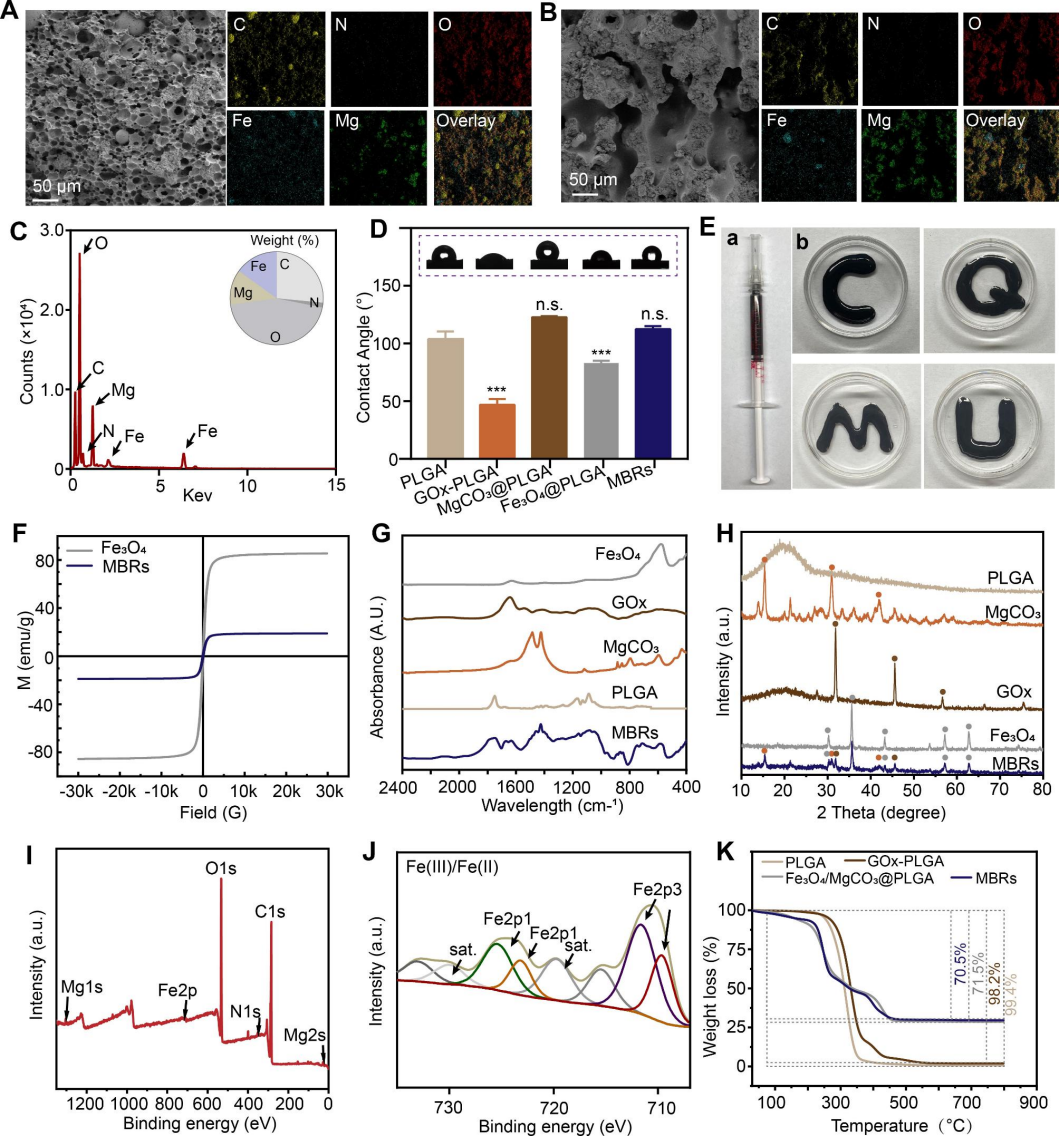
Fabrication and characterization of various forms of MBRs: Fe_3_O_4_/GOx/MgCO_3_@PLGA gels. SEM images and corresponding mapping images of MBRs without (**A**) and with (**B**) AMF exposure. (**C**) Energy spectrum of MBRs (insert figure showing weight% analysis of elements). (**D**) WCA test results of different groups (insert: optical images). (**E**) Digital images of the injectable MBRs through a standard 1 ml syringe and representative images of different forms. (**F**) Magnetic hysteresis loop of MBRs and Fe_3_O_4_ nanoparticles. (**G**) FTIR spectra of PLGA, MgCO_3_, GOx, Fe_3_O_4_ and MBRs. (**H**) XRD spectra of PLGA, MgCO_3_, GOx, Fe_3_O_4_ and MBRs. (**I**) XPS spectra of MBRs. (**J**) Fe 2p XPS of MBRs. (**K**) TGA curves of PLGA, GOx-PLGA, Fe_3_O_4_/MgCO_3_@PLGA and MBRs.

The SEM images and corresponding mapping photographs further verified a uniform porous structure with a diameter less than 50 μm and homogeneous distributions of C, N, O, Fe and Mg for most MBRs (Fig. 1A). Moreover, after AMF exposure, the internal morphology of the MBRs exhibited an increased porous “bridge-like” structure with favorable stable element dispersion (Fig. 1B). The corresponding element quantitative result by energy spectrum is shown in Fig. 1C. In addition, the porosity of the MBRs was 40.3 ± 2.3% without AMF application, while it increased to 68.9 ± 4.2% after AMF exposure, indicating that GOx could be released into tumors by AMF triggering. As shown in Fig. 1D, after liquid‒solid transformation, the MBRs displayed no difference from PLGA in terms of hydrophilicity, which indicated that there was no effect on cell adhesion. For irregular bone defect repair biomaterials used in a minimally invasive strategy, good injectability and shape adaptability are vital. The liquid form of MBRs were taken into a standard syringe (Fig. 1E-a). It was found that the MBRs could freely pass through the syringe due to their low viscosity and form various irregular surface profiles (Fig. 1E-b). For use in magnetic hyperthermia, a satisfactory magnetic property is necessary [27, 28]. As the long and narrow shaped hysteresis curve (Fig. 1F) shows, the MBRs are soft magnetic ferrite and have a low coercive force and residual magnetization value (saturation magnetization: 18.92 emu/g), similar to those of pure Fe_3_O_4_ nanoparticles (saturation magnetization: 85.52 emu/g). As a soft magnetic material with relatively low hysteresis, loss properties and coercive force, MBRs can be easily triggered by an AMF because the coercive force is smaller than that of the AMF [29]. ICP-OES quantitative measurement shown that the iron concentrations of MBRs in the first batch with and without heating were 108.196 ± 1.097 mg/g and 108.516 ± 0.6 mg/g, respectively, indicating no significant difference between the two groups (p > 0.05) and illustrating that no Fe_3_O_4_ nanoparticles escaped from the MBRs after heating.

To further determine the composition and chemical construction elemental status of the MBRs, several test analyses of the as-prepared solid samples were performed. The FTIR spectrum of MBRs indicated the characteristic absorption peaks of MgCO_3_ and GOx, confirming the presence of MgCO_3_ and GOx molecules (Fig. 1G). Moreover, the results of the X-ray diffraction (XRD) assay (Fig. 1H) suggested that the chemical structure of the incorporated Fe_3_O_4_ and MgCO_3_ would not be changed by the preparation and liquid‒solid phase transition process of the MBRs. Notably, the presence of the elements C, N, O, Mg, and Fe was confirmed by X-ray photoelectron spectroscopy (XPS) analysis associated with C 1s, N 1s, O 1s, Mg 2s, and Fe 2p, respectively (Fig. 1I). The C 1s spectrum of the C-O peak at 532.5 eV (Figure S4) is in contrast with the C 1s spectrum of the MBRs, which shows a new higher C-H peak at 284.6 eV (Figure S5), attributed to the C-H of the GOx particles. In addition, the characteristic Mg 1s peak at 1034.5 eV of the shakeup satellite peaks corresponding to that of Mg^2+^ was observed from the high-resolution Mg 1p XPS spectrum of the MBRs (Figure S6). Compared to the solid form of PLGA, the emerged characteristic Fe 2p peaks at 710.5 eV (Fe 2p3) and 725.9 eV (Pt 2p1) were attributed to that of Fe^2+^ and Fe^3+^, which was caused by the modification of the Fe(III)/Fe(II) prodrug (Fig. 1J). The presence of Mg and Fe indicated high potential for triggering magnetic hyperthermia and Mg-related bone repair. Furthermore, after the heating process (from 37 ℃ to 800 ℃), the residual composition in the various scaffolds was estimated, as shown in Fig. 1K. The MBRs showed good thermal stability under heating conditions, and no obvious thermal decomposition of the material was observed in the magnetothermal working temperature range (< 50 ℃). The significant weight loss observed for Fe_3_O_4_/MgCO_3_@PLGA and the MBRs upon heating was ascribed to the thermal decomposition of MgCO_3_. Compared with Fe_3_O_4_/MgCO_3_@PLGA, the approximately 1% weight loss of the MBRs was consistent with the loading dose of GOx. Collectively, these good performances of the MBRs indicated that MgCO_3_, Fe_3_O_4_ and GOX were successfully loaded into the PLGA gels.

### Magnetic hyperthermia characteristics of MBRs

**Fig. 2:**
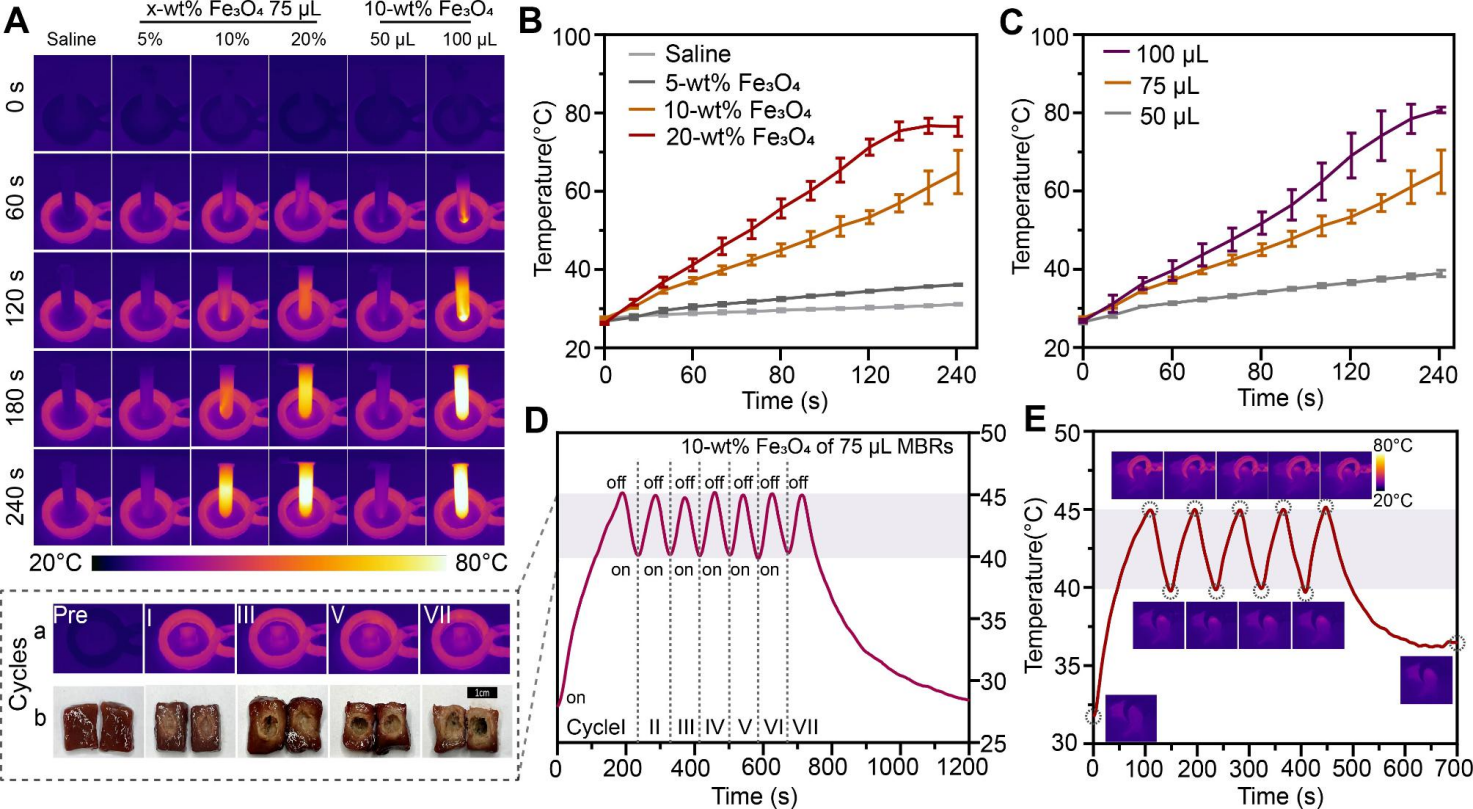
Mild magnetothermal performance of various forms of MBRs-Fe_3_O_4_/GOx/MgCO_3_@PLGA gels in vitro and in vivo. (**A**) In vitro infrared thermal images of Fe_3_O_4_/MgCO_3_@GOx-PLGA with different volumes and various mass fractions of Fe_3_O_4_ nanoparticles. (**B**) Corresponding quantitative temperature curves of saline and MBRs (incorporated with different mass fractions of Fe_3_O_4_ nanoparticles). (**C**) Corresponding quantitative temperature curves of 10% Fe_3_O_4_-MBRs with various volumes. (**D**) Quantitative temperature curves of 75 µL 10% Fe_3_O_4_-MBRs for “on-off” model magnetothermal in vitro bovine liver treatment with corresponding infrared thermal images and digital images of several cycles. (**E**) Temperature-time curve of 143B tumor-bearing mice after intratumoral injection of MBRs and AMF exposure for five on-off cycles and the corresponding infrared thermal images

To achieve controllable ranges of heating temperatures and mild magnetothermal effects in vivo, the optimal Fe_3_O_4_ mass ratio needs to be accurately evaluated. The results of magnetothermal tests indicated that the presence of MBRs could quickly and effectively increase the temperature of the surroundings according to the infrared thermography images (Fig. 2A) and corresponding temperature curves (Fig. 2B). The saline solution without magnetic materials showed no significant temperature variations. For Fe_3_O_4_-MBRs loaded with various mass ratios (10%, 20%), the hyperthermia temperature easily reached over 45 °C, while the 5% Fe_3_O_4_-MBRs did not exhibit this performance. For the 20% Fe_3_O_4_-MBRs, the final temperature of the surrounding water bath was over 80 °C. As volume of 10% Fe_3_O_4_-MBRs increased, the magnetothermal effect improved (Fig. 2C). However, for 100 µL of 10% Fe_3_O_4_-MBRs, the temperature control property was not ideal due to its steep temperature curve, which exceeded 80 °C and was not suitable for mild magnetothermal therapy. Hence, 75 µL of 10% Fe_3_O_4_-MBRs was optimal for further experiments. To simulate the intratumor therapeutic range to avoid injuring surrounding healthy tissue, the ex vitro bovine liver was used to evaluate the treatment effect.

Temperatures in the range of 40 ~ 45 °C can not only eradicate cancer cells directly with little damage to surrounding normal tissue but also induce combination therapy, especially for GOx as an enzyme compound retaining protein activity [30, 31]. Hence, we attempted to apply an “on-off” cyclic heating model that maintained the target temperature in an effective range [32]. As Fig. 2D-a shows, the 75 µL of 10% Fe_3_O_4_-MBRs presented good magnetic thermal stability during the thermal process, and the temperature of the center of the bovine liver persistently and mildly increased. After several cycles of magnetic thermal heating, the morphology of the bovine livers and the ablation range were measured, showing livers with pale tissue and obvious coagulative necrosis under a microscope after treatment (Fig. 2D-b). For the five heating cycles, the therapeutic range was approximately 1 cm, which is the best fit for use in tumors (Figure S7). Moreover, the exposure duration was selected based on the tumor volume, which potentially offers an individual treatment scheme for patients with various tumor sizes by varying the exposure duration and implanted content of MBRs. The “on-off” cyclic heating strategy is also appropriate for application in vivo (Fig. 2E). In 143B tumor-bearing mice, the MBRs also exhibited good magnetothermal stability during the heating process. Consequently, 75 µL of 10% Fe_3_O_4_-MBRs was chosen in this work as the optimal mild MTT condition for triggering GOx release, and it is a rational choice for subsequent MTT and starvation therapy.

### Mild hyperthermia-triggered GOx release for inducing starvation therapy

**Fig. 3:**
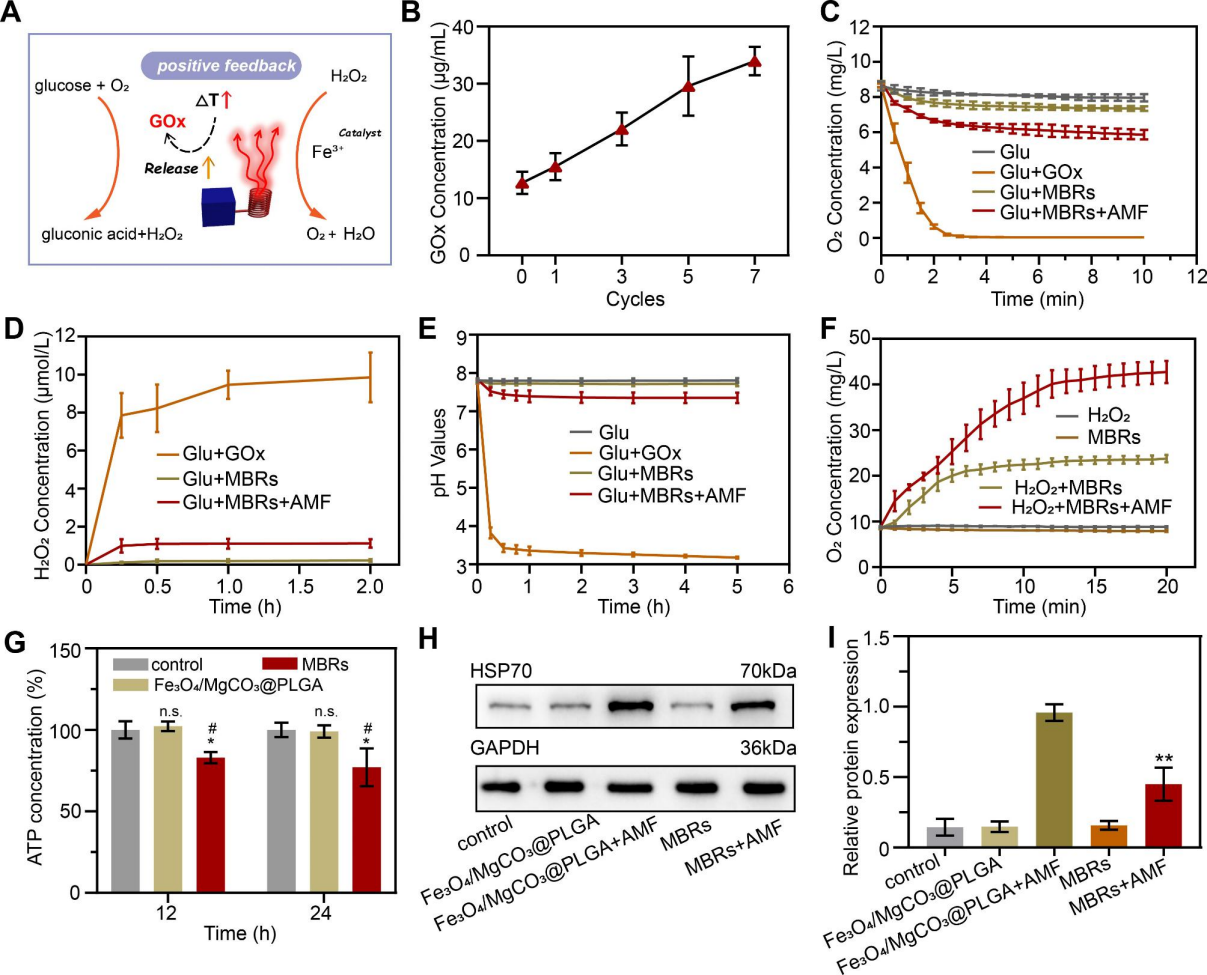
Mild hyperthermia-triggered GOx release to induce starvation therapy. (**A**) Schematic illustration of the mechanism of the mild hyperthermia-triggered GOx enzymatic reaction. (**B**) Release of GOx from MBRs after AMF exposure. (**C**) Oxygen concentration changes over time in different solutions of glucose, GOx + glucose, MBRs + glucose, and MBRs + AMF + glucose. (**D**) Changes in H_2_O_2_ concentration resulting from reactions with GOx + glucose, MBRs + glucose, and MBRs + AMF + glucose at various time points. (**E**) Changes in pH resulting from the reaction between glucose, GOx + glucose, MBRs + glucose, and MBRs + AMF + glucose at various time points. (**F**) Time-dependent curves of oxygen concentration for H_2_O_2_, MBRs, H_2_O_2_ + MBRs, and H_2_O_2_ + MBRs + AMF. (**G**) ATP concentration of 143B cells from different groups after AMF exposure. (**H**) Representative immunoblot results of different protein levels of HSP70 for each treatment group and (**I**) corresponding quantitative analyses of the ratios of HSP70/GAPDH. (The data are shown as the means ± SDs, n = 3 per group, n.s. represented no significance and *p < 0.05, **p < 0.01, in comparison with the control groups, ##*p* < 0.001 in comparison with Fe_3_O_4_/MgCO_3_@PLGA, respectively.)

To explore the mechanism by which mild hyperthermia triggers GOx release to induce starvation therapy, the GOx-mediated reaction process and the mutual promoting effect need to be further verified. As expected, mild magnetic hyperthermia enhanced the release of GOx by the MBRs to accelerate the transformation of glucose (Glu) into gluconic acid and H_2_O_2_, and H_2_O_2_ could further transform into O_2_ and H_2_O, especially in the thermal environment (Fig. 3A). When GOx is used as an antitumor drug in biomaterials, we expect it to play a tumor killing role and reduce the impact on surrounding normal cells through controlled release. According to the standard curve of the GOx solution (Figure S8), the cumulative release concentration of GOx from the MBR + AMF group was much higher than that from the MBR group (Figure S9), which means that triggered GOx release was achieved by the AMF. Furthermore, the amount of GOx release was positively related to “on-off” treatment cycles (Fig. 3B), which illustrated that MTT was a driver of MBRs for the GOx reaction. The oxygen concentration was measured by a portable dissolved oxygen meter to observe the GOx-mediated oxygen consumption ability [33]. According to the time-dependent curves in Fig. 3C, the concentration of oxygen in the Glu + GOx group rapidly decreased as the reaction continued and reached a plateau in 3 min, while the Glu group displayed almost no reaction. This result indicated that soluble oxygen could be produced through GOx-mediated glucose oxidization. In addition, because GOx was encapsulated in the MBRs, the oxygen content of the Glu + MBRs group decreased more slowly than that of the Glu + GOx group, i.e., free GOx solution. Moreover, due to the release of activated GOx from the MBRs, the oxygen concentration in the Glu + MBRs + AMF group decreased more quickly than that in the Glu + MBRs group, which implied that MTT is a positive promoter of the GOx-mediated starvation reaction. However, compared with the Glu + GOx group, the oxygen concentration in the Glu + MBRs + AMF group decreased slowly, demonstrating the reoxygenation capacity of MBRs + AMF by thermal decomposition or by Fe^3+^ catalyzing H_2_O_2_ to O_2_ and indicating that this strategy could accelerate substrate consumption for propulsive reactions.

The same principles were applied for detecting substrate production. The oxygen concentration in the Glu + GOx + H_2_O_2_ group increased quickly within 15 min, while negligible changes were detected in the Glu + MBRs + AMF + H_2_O_2_ groups (Fig. 3F). Moreover, with the production of gluconic acid, the pH continued to decrease [34]. As the starvation reaction progressed, the H_2_O_2_ and gluconic acid concentrations gradually increased (Fig. 3D and E), which confirmed that the controlled release of GOx was achieved by triggering the MBRs through MTT, effectively catalyzing the glucose oxidation reaction and converting glucose into gluconic acid and H_2_O_2_. As Fig. 3F shows, in the H_2_O_2_ + MBR + AMF group, H_2_O_2_ decomposed into H_2_O and O_2_ under the mild thermal treatment as the reaction occurred, and Fe^3+^ decomposed from Fe_3_O_4_ under the AMF to facilitate H_2_O_2_ dissociation. Without AMF triggering, the amount of H_2_O_2_ decomposition markedly decreased. These results further confirmed that MTT could be beneficial for the progression of GOx-mediated starvation reactions.

The positive feedback of mild hyperthermia–starvation mutual synergistic therapy may also involve a thermoresistance remission mechanism. Previous studies have shown that HSPs produced in the heat reaction process can cause tumor heat resistance, and this pathway is very dependent on ATP as an energy supply [35, 36]. Therefore, delaying ATP production is expected to overcome the heat resistance of tumors and enhance the therapeutic effect of MTT. As expected, a relatively low ATP concentration was detected after treatment with MBRs after AMF irradiation (Fig. 3G), presumably because the starvation reaction consumes glucose. HSP70 expression in 143B cells under different treatments was evaluated by Western blot (WB) analysis. According to the predicted results, high HSP70 expression was observed in 143B cells treated with Fe_3_O_4_/MgCO_3_@PLGA after AMF exposure, directly indicating a defensive heat shock response via thermal therapy (Fig. 3H). The MBRs + AMF treatments significantly decreased the HSP70 expression levels, and the results were consistent with ATP concentration variation, verifying that HSP overexpression could be inhibited by interfering with ATP production. After the intervention of 143B cells with different treatments, the HSP70 levels were measured. After Fe_3_O_4_/MgCO_3_@PLGA + AMF treatment, the HSP70 level increased 3.82-fold. In contrast, the MBRs + AMF group significantly reduced the relative level of HSP70 by 1.95-fold (Fig. 3I). Taken together, these results suggest that GOx could be an effective adjuvant for enhancing MBRs-based MTT by downregulating HSP expression.

### Efficacy of mutually synergistic mild hyperthermia–starvation therapy in vitro

**Fig. 4:**
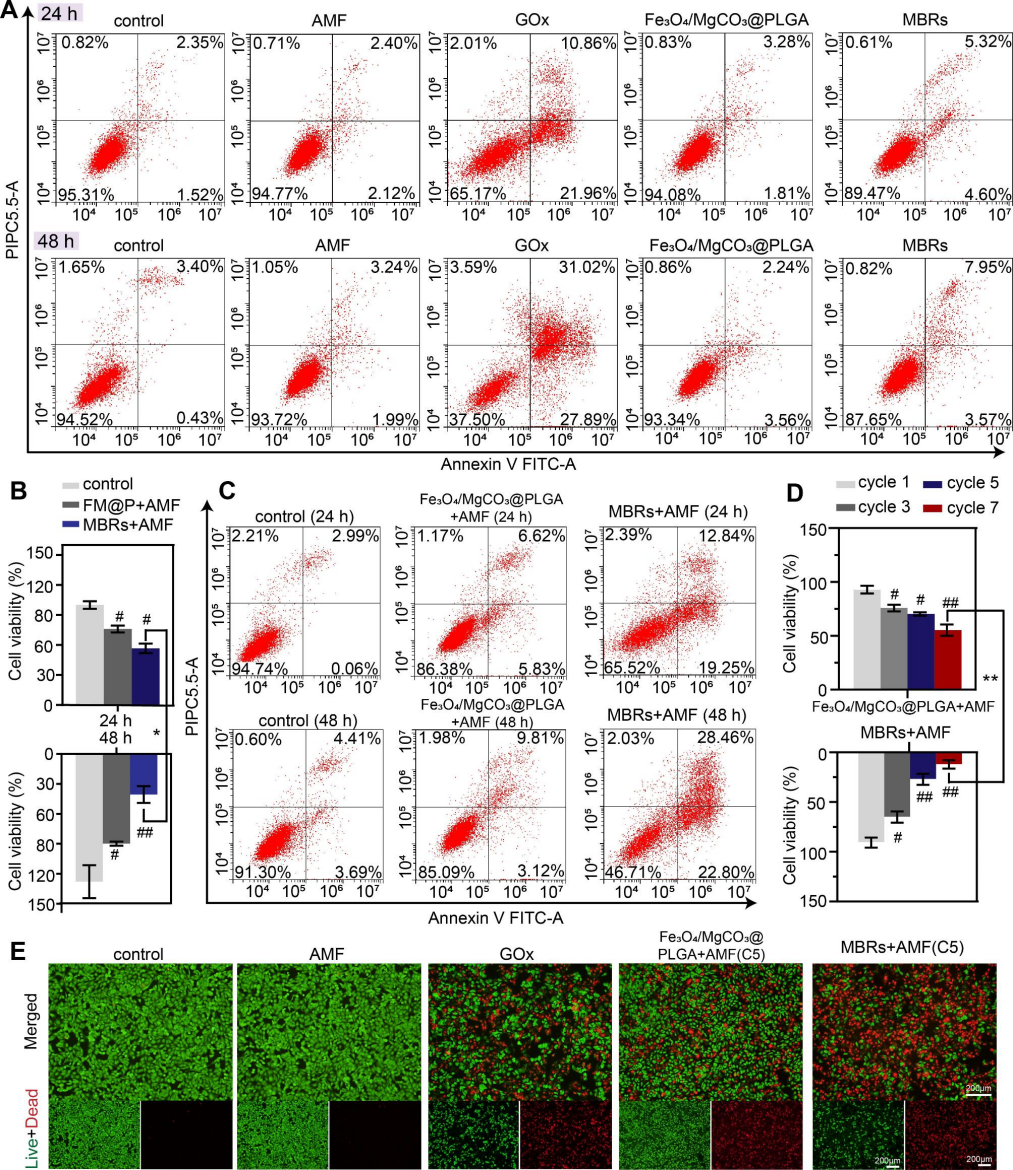
In vitro mild hyperthermia-starving mutual synergistic therapy against 143B cells. (**A**) Flow cytometry was used to determine and analyze the apoptosis of 143B cells cocultured with PBS, AMF, GOx, Fe_3_O_4_/MgCO_3_@PLGA, and MBRs for 24 and 48 h. (**B**) Cell viability assay and (**C**) flow cytometry analysis of 143B cells incubated with PBS, Fe_3_O_4_/MgCO_3_@PLGA and MBRs after AMF exposure for 24 and 48 h. (**D**) Cell viability assay results of 143B cells after various cycles of “on-off” model magnetothermal treatment for Fe_3_O_4_/MgCO_3_@PLGA and MBRs groups. (**E**) Calcein-AM (green)/PI (red) staining images of 143B cells after various treatments (scale bars: 200 μm). (The data are shown as the means ± SDs, n = 3 per group, n.s. represents no significance and #*p* < 0.05, ##*p* < 0.01, in comparison with the control groups, *p < 0.05 **p < 0.01 in comparison between groups, respectively.)

Good biocompatibility and biosafety are prerequisites of responsive functionalized implants for in vivo applications, and the harmlessness of the MBRs without using an AMF was expected. Therefore, the biocompatibility and biosafety of the MBRs and their therapeutic effects were examined by cell apoptosis using flow cytometry. For GOx solution without PLGA gel, the percentage of viable cells showed a sharp decline of 62.5% in coculture for 48 h. However, the MBRs showed high biosafety without AMF exposure when cocultured with cells for 24 and 48 h, indicating the harmlessness of the MBRs with PLGA gels. Compared to the MBRs group, the concentration of GOx in the GOx group was higher when GOx was directly added into the tumor cell culture system. The excessive GOx rapidly consumed glucose in the culture medium and produced H_2_O_2_, inhibiting tumor cell metabolism and increasing oxidative stress levels within the tumor cells, ultimately killing tumor cells, which might cause a sharp decrease in blood sugar and interfere with normal cell metabolism, and is not suitable for in vivo treatment. However, when combined with AMF triggering, the cell viability declined under coincubation with Fe_3_O_4_/MgCO_3_@PLGA and MBRs. In contrast, the Fe_3_O_4_/MgCO_3_@PLGA group’s cytotoxic efficacy was much lower than that of the MBRs groups with the same treatment and irradiation time as the CCK-8 assay (Fig. 4B) and flow cytometry analysis (Fig. 4C). The GOx release triggered by magnetic hyperthermia and the mutually synergistic hyperthermia–starviation therapy effect could explain this result.

As expected, after it was determined that the MBRs have superb magnetic thermal properties and the ability to trigger GOx release to induce starvation therapy, their antitumor efficacy under AMF exposure was investigated as another important property. As mentioned above, as the number of treatment cycles increased, the in vitro magnetic hyperthermia effect improved. Cell viability was further evaluated with a CCK-8 assay, and a similar result was obtained. For the MBR group, after seven cycles of AMF exposure, the survival rate of the cells was markedly decreased and almost converged to zero after 48 h (Fig. 4D). To prevent normal tissue damage, the five-cycle “on-off” strategy was deemed suitable for further experiments, achieving similar efficacy to that of in vitro magnetic hyperthermia. The inhibitory effect of the MBRs on 143B cells was visually examined using a calcein-AM/PI staining assay. Live cells (green fluorescence staining) and dead cells (red fluorescence staining) were observed by fluorescence microscopy. The control and AMF-only groups exhibited no red fluorescence, indicating that an AMF has no cell killing ability toward 143B cells. More pronounced red fluorescence was observed in the GOx solution group, indicating that the predominant cell-killing effect of GOx was independent of AMF triggering, which might be relevant to preventing unintended injury to normal cells (Fig. 4E). More importantly, as Fig. 4E and Figure S10 show, the MBRs incorporated with GOx produced strong red fluorescence after five cycles of treatment, showing an enhanced but significantly similar therapeutic effect to that of Fe_3_O_4_/MgCO_3_@PLGA. These results demonstrated that mutual synergistic hyperthermia**–**starvation therapy had an excellent effect against 143B cells.

### Antitumor therapeutic efficacy and biosafety of the MBRs

**Fig. 5:**
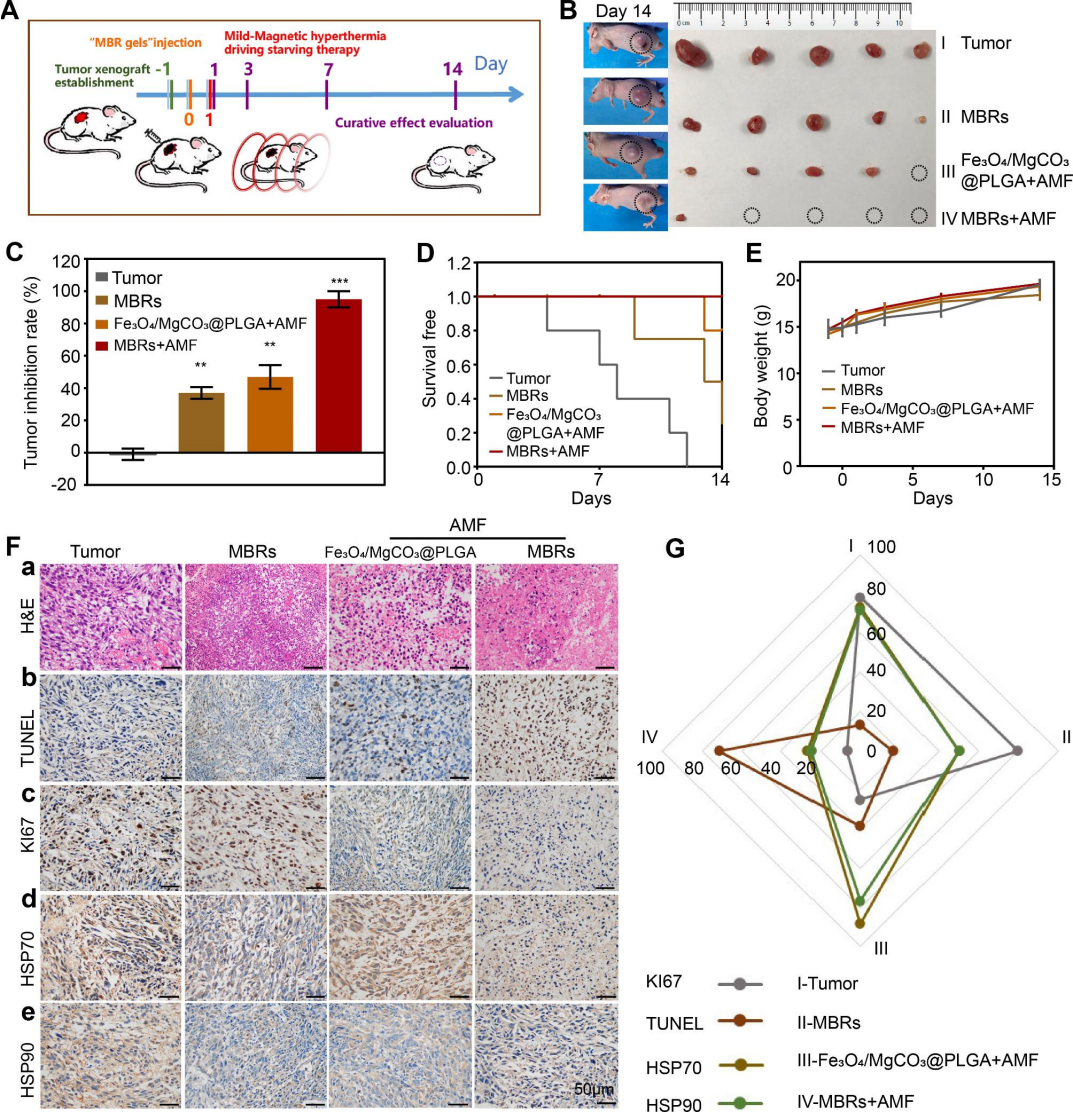
Antitumor therapeutic efficacy of MBRs. (**A**) Treatment and follow-up regimen. (**B**) Picture of the excised 143B tumors 14 days after treatments and the corresponding tumor-bearing mice. (**C**) Tumor inhibition rate of 143B tumor-bearing mice with the various treatments. (**D**) Morbidity-free survival and (**E**) body weight of 143B tumor-bearing mice in the different treatment groups. (**F**) H&E staining (a) and immunohistochemical staining (TUNEL-b, Ki67-c, Hsp70-d and Hsp90-e) in the tumor region of each group. (**G**) Quantitative analysis of immunohistochemical staining (Fig. 5-F). (Scale bars: 50 μm). (The data are shown as the means ± SDs, n = 5 per group, n.s. represented no significance and *p < 0.05, **p < 0.01, ***p < 0.001 in comparison with the control groups, respectively.)

Considering the predominant curative effect in vitro and the good solid transformation performance of the MBRs in tumor tissues, we anticipate that MBRs could function as triple-functional magnetic gels for in vivo osteosarcoma therapy. Following therapeutic process described in Fig. 5A, we established a 143B human osteosarcoma tumor-bearing nude mouse model and stochastically divided the mice into 4 groups: Tumor group (PBS), MBR group (MBRs), Fe_3_O_4_/MgCO_3_@PLGA with AMF exposure group (Fe_3_O_4_/MgCO_3_@PLGA + AMF), and MBRs with AMF exposure group (MBR + AMF). The mice were injected with different gels (75 µL per mouse), followed by AMF irradiation (five cycles). Because MBRs provide superb temperature control, the tumor-site temperature in the Fe_3_O_4_/MgCO_3_@PLGA + AMF group and the MBRs + AMF group was accurately increased to 40–45 °C during the exposure cycles (Fig. 2E). After treatment with different interventions, the volume of the tumors and the body weights were calculated at different time points (Fig. 5A). As the tumor image (Fig. 5B) and inhibition rate (Fig. 5C) shown, the tumors in the saline-treated group grew rapidly (Tumor group-control), with a maximum tumor volume over 1 cm^3^. For treatment with MBRs alone, the tumor growth inhibitory effect was unsatisfactory (inhibition rate: 37 ± 3.6%). This was also the case for mice treated with Fe_3_O_4_/MgCO_3_@PLGA plus AMF exposure (inhibition rate: 46.9 ± 7.4%). Because the amount of GOx released by the MBRs was small, the efficiency of GOx-mediated glucose depletion was low, and using only magnetic hyperthermia led to residual tumor. In comparison, a marked inhibition of tumor size was observed in the MBRs + AMF group (inhibition rate: 95 ± 5%), which is due to the effect of mutually synergistic mild hyperthermia**–**starvation mutual synergistic therapy. The morbidity-free survival from each treatment group further confirmed the above results (Fig. 5D). Moreover, no significant body-weight variations were observed in each group (Fig. 5E), indicating that each functional particle of MBRs had high biosafety and biocompatibility for in vivo applications.

To further evaluate the substantial damage of different groups to tumor cells and the corresponding potential mechanisms, H&E, KI67, and TUNEL staining were performed. In H&E staining, the combination of MBRs with AMF irradiation caused the most significant large-area cell shrinkage and severe necrosis (Fig. 5F-a). Only Fe_3_O_4_/MgCO_3_@PLGA + AMF resulted in moderate cell necrosis, while no obvious cell damage was found in the other two groups. The above cell morphology indicated that different therapeutic strategies produced different therapeutic effects. Immunohistochemistry (IHC) staining was also carried out to evaluate the apoptotic index and proliferative index after different treatments. The most distinct 143B cell apoptosis, as observed by TUNEL staining of the nucleus (Fig. 5F-b), was observed after treatment with MBRs combined with AMF irradiation, which also shown the greatest inhibitory effect on proliferation, as shown by KI67 staining (Fig. 5F-c).

To further illustrate the underlying mechanism of synergistic therapy, IHC staining of HSP70 and HSP90 was used to investigate the inhibitory effect of MBRs-mediated mutual synergistic hyperthermia**–**starvation therapy on these heat-resistant proteins. As shown in Fig. 5F-d and **e**, the expression levels of HSP70 and HSP90 in the Fe_3_O_4_/MgCO_3_@PLGA + AMF group were much higher in the cytoplasm, as indicated by dark brown staining, than those in the MBRs group, revealing that the expression of HSP70 and HSP90 may be induced by magnetic hyperthermia. However, the IHC staining intensity results of the MBR + AMF group were obviously lower than those of the Fe_3_O_4_/MgCO_3_@PLGA + AMF group because magnetic hyperthermia triggered the GOx release-mediated starvation effect through reduced ATP generation to inhibit HSP expression. Moreover, the IHC intensities of HSP70 and HSP90 were analyzed, and the results were the same (Fig. 5G).

To evaluate the possibility of clinical MBRs transformation, the biocompatibility of the MBRs was evaluated with regard to their potential use as therapeutic agents. First, the H&E staining diagram (Figure S11) and organ weight (Figure S12) of the major organs (including the heart, liver, spleen, lung and kidney) of the MBRs-treated mice showed no obvious abnormalities or tissue damage, indicating the negligible toxicity of the MBRs to the main organs. Moreover, blood samples were collected from mice after various treatments to evaluate the corresponding systemic toxicity (Figure S13). Compared with those in the control group, none of the hematological parameters in the MBR group were significantly changed at 7 and 28 days after injection. These results suggested that MBRs are highly biocompatible as promising therapeutic agents for mutual synergistic hyperthermia**–**starvation therapy against 143B OS in vivo.

### MBRs accelerated osteogenesis in vitro and in vivo

**Fig. 6:**
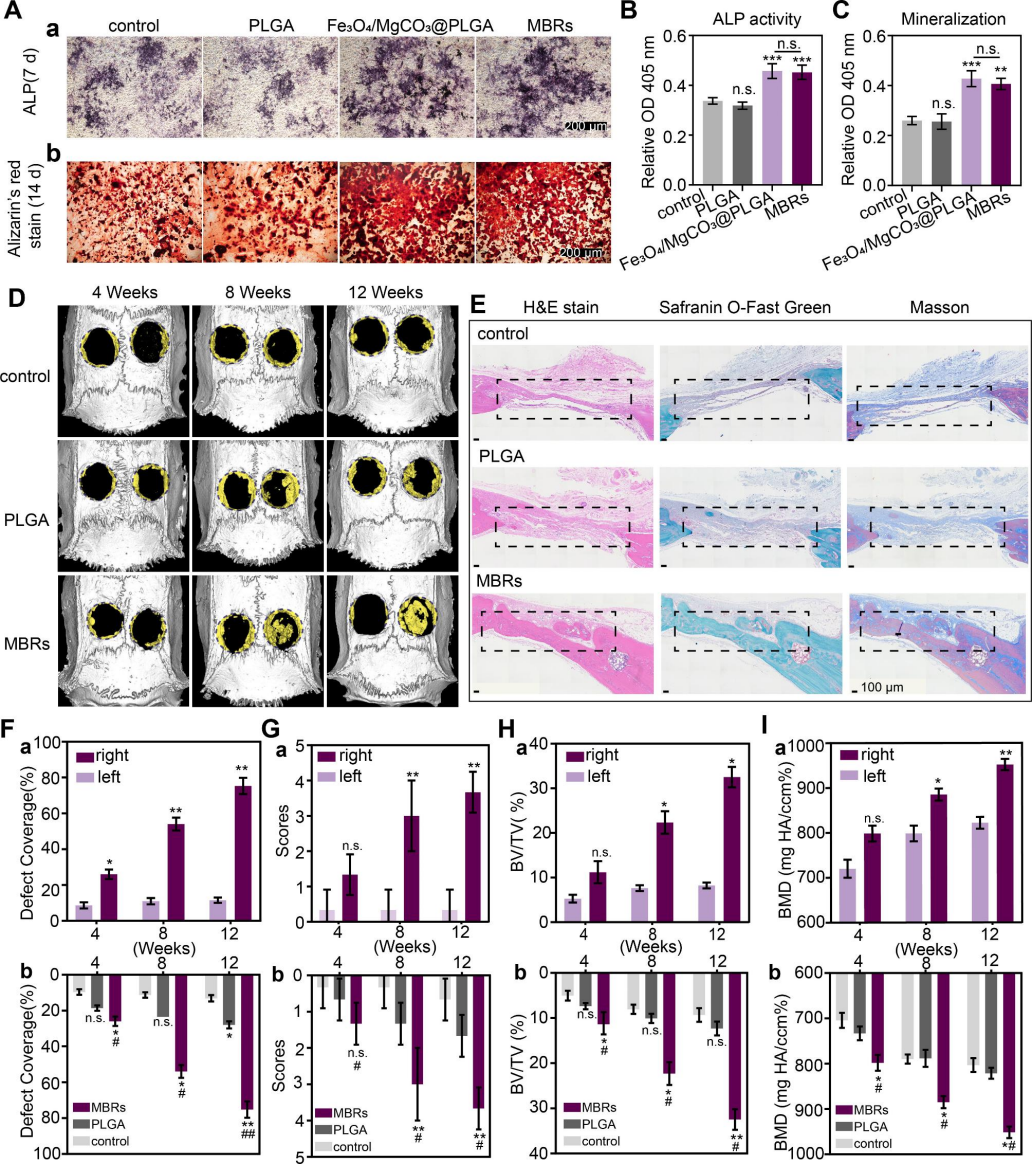
Evaluation of the MBRs ability for bone regeneration in vitro and in vivo. (**B**A) ALP staining and alizarin red staining (ARS) of PBS, PLGA, Fe_3_O_4_/MgCO_3_@PLGA and MBRs. Quantitative analysis of (**B**) ALP activity and (**C**) ARS staining. (**D**) Reconstructed 3D micro-CT images of rat crania with the treated defects (left: self-blank control, right: treatment with different groups). (**E**) Histological evaluation of bone defect regeneration by H&E, safranin O-fast green and Masson’s trichrome staining (the repair area is labeled with rectangular boxes, scale bars: 100 μm). Quantitative analysis of bone defect regeneration as determined by (**F**) the defect coverage percent, (**G**) the score of bone defect restoration, (**H**) BV/TV% and (**I**) BMD: (a) within the MBRs group and (b) among the three groups. (The data are shown as the means ± SDs, n = 3 per group, n.s. represented no significance and *p < 0.05, **p < 0.01 in comparison with the control groups, # *p* < 0.05 ## *p* < 0.01 in comparison with PLGA groups, respectively

Considering the bioactive effects of magnesium ions, such as therapeutic osteoinductive functions and enhancing matrix mineralization, MgCO_3_ was loaded into PLGA gels as a Mg^2+^ source to obtain Fe_3_O_4_/GOx/MgCO_3_@PLGAgels (MBRs). Additionally, the osteogenic peculiarities of MBRs were evaluated in vitro and in vivo. ALP staining on day 7 implied the distinguished osteogenic differentiation of iMEFs in the Fe_3_O_4_/MgCO_3_-PLGA group and MBRs group compared with the control group and PLGA group (Fig. 6A-a). This is most likely because the Fe_3_O_4_/MgCO_3_-PLGA group and the MBRs group both exhibited sustainable Mg^2+^ release. Furthermore, quantitative analysis shown that ALP activity was also significantly higher than that in the other groups, indicating that MBRs promoted the osteogenic differentiation of iMEFs, while PLGA gels alone did not show this property (Fig. 6B). In addition to the improved ALP production, ARS staining was used to directly visualize mineralization **(**Fig. 6A-b**)**, which formed at the late stage of osteogenic differentiation and was assessed by quantitative analysis **(**Fig. 6C**)**, revealing an obvious increase in mineral deposition induced by the MBRs. Hence, MBRs have the greatest capacity to promote the osteogenic differentiation of iMEFs in vitro.

As designed, MBRs with good injectability and shape adaptability were injected into established cranial defects and stabilized after phase transformation. The right side was the treatment group, and the left side was used for the self-control study. After twelve weeks of implantation, more freshly generated osteogenic tissue was observed in the MBRs group than in the PLGA group, as determined by micro-CT (Fig. 6D); however, there was hardly any new bone in the control group. Additionally, the representative H&E, Masson and safranin O-fast green staining images of 12-week-old rat calvarial bones showed much more new bone and mineralized osseous components on the right side of the MBRs group than in the other two groups, which was in accordance with the micro-CT results (Fig. 6E). Moreover, in the MBRs group, a complete bone bridge with a thick structure and plenty of marrow spaces was eventually formed, suggesting the occurrence of bone remodeling. According to the micro-CT scanning results, the quantitative indexes were evaluated to measure the quality and quantity of the newly formed bone tissue. As Fig. 6F shows, the defect coverage of the MBRs group was significantly superior to that of the other groups 12 weeks post-operation. With time, the right side of the MBRs group showed new bone, while the left side did not, indicating the bone reconstruction by MBRs has good stability in terms of location. The degree of bony bridging and bone healing based on micro-CT images was assessed by the guideline score. Bony bridges of the entire defect span were observed at the widest point in the MBRs group (Fig. 6G), whereas only a few scattered bone spicules at the defect boundary were observed in the PLGA group and the control group. Moreover, the bone volume/total volume (BV/TV) and BMD values indicated the improved osteogenic functionality and mineralization of the MBRs group (Fig. 6H and I) compared to the other groups. For the MBRs group, a comparison between the defects of the unfilled side (left) and the filled side (right) was consistent with the above results. The data showed significant new bone formation in the MBRs group. In conclusion, it was confirmed that MBRs have a significant osteogenic function, which can not only promote bone regeneration but also assist bone mineralization, which is very beneficial for the repair of bone defects of in OS.

### Anti-residual bone tumor effect of MBRs in situ

**Fig. 7:**
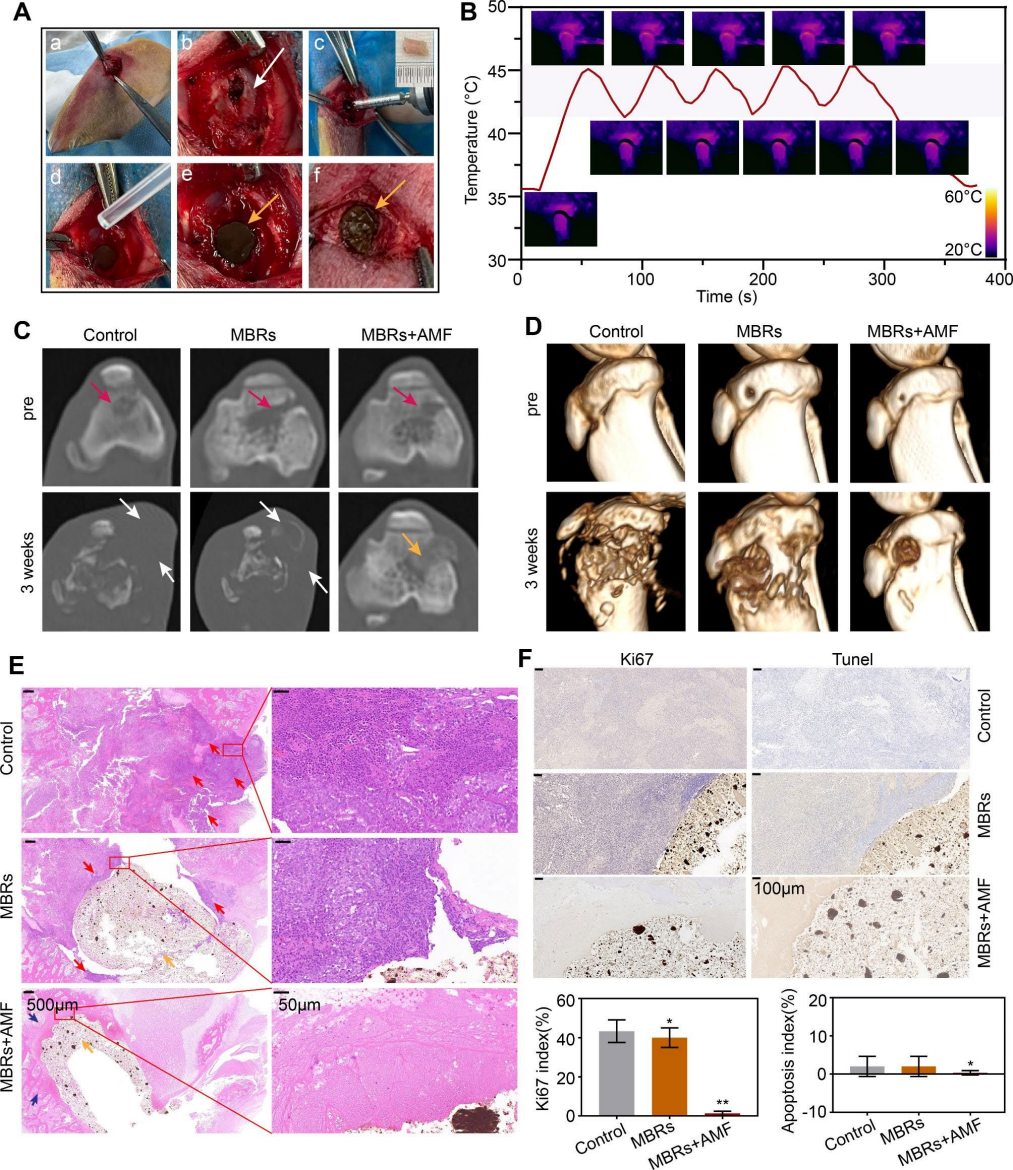
Evaluation of the MBRs anti-tumor ability for bone tumor in situ. (**A**) The processes of establishment of a residual tumor model after surgical treatment of tibial plateau bone tumors in rabbits (white arrow: postoperative bone defect, yellow arrows:MBRs). (**B**) Temperature-time curve of rabbit leg in MBRs group with AMF exposure for five on-off cycles and the corresponding infrared thermal images. (**C**) Axial position CT images at each follow-up time point of each group (red arrow: bone tumor in situ, white arrow:bone destruction and swelling of soft tissue, yellow arrow: injected solid form MBRs). (**D**) 3D-reconstructed CT images at each follow-up time point. (**E**) Histological evaluation of bone defect and bone tumor after treatment (scale bar is 500 μm and 50 μm, red arrow: bone tumor in situ, blue arrow: bone tissue after treatment, yellow arrow: injected solid form MBRs). (**F**) Immunohistochemical staining (Tunel and Ki67) in the tumor region of each group and corresponding quantitative analysis (Scale bars: 100 μm). (The data are shown as the means ± SDs, n = 5 per group, n.s. represented no significance and *p < 0.05, **p < 0.01 in comparison with the control groups, respectively.)

MBRs have shown a good antitumor effect on 143B human osteosarcoma in a previous study, as well as excellent osteogenic effects in rat skull defect models. However, to achieve the clinical translation of MBRs, it is still necessary to further explore the treatment of tumors by simulating the anatomical environment of bone tumors. Currently, in clinical practice, surgical resection is the first choice for treating tumors that occur in load-bearing bones; however, surgery can only remove tumor tissues visible to the naked eye. Therefore, multifunctional MBRs not only exert anti-OS effects in a minimally invasive manner but also assist in inhibiting the growth of residual tumors after surgical treatment, which has important significance. We prepared a rabbit tibial plateau residual tumor model in situ, as shown in **Fig. 7Aa-c**. The tumor tissue was partially removed with bone. H&E staining showed that tumor tissue was distributed around cancellous bone, indicating that only part of the tumor tissue was removed during surgery and that some tumor tissue remained in the tibial plateau (Figure S14). Subsequently, liquid MBRs were injected into the defect and became solid through liquid exchange with surrounding tissues. Surprisingly, MBRs could not only completely fill the defect but also inhibit bone tissue bleeding through liquid exchange, reducing the amount of bleeding during surgery (Fig. 7**Ad-f**). Then, the tumor site in the rabbit leg was subjected to five mild “on-off” cycles of magnetothermal treatment, and the results indicated that the filled MBRs were evenly heated and had stable magnetothermal properties (Fig. 7B).

To evaluate the growth of tumors in the tibial plateau, each group of rabbits underwent CT scanning before the intervention and three weeks after treatment. As Fig. 7C shows, the residual tibial plateau tumors in both the untreated group and the MBR group recurred after 3 weeks, with visible bone destruction and surrounding soft tissue swelling (white arrows). However, in the MBR + AMF group, there was no bone destruction or soft tissue damage around the tibial plateau. The CT reconstructions (Fig. 7D) showed similar results, with significant bone destruction in the control and MBR groups. H&E staining showed a large area of uniformly red-stained cytoplasm, indicating disordered tissue and marked cellular destruction in the MBR + AMF group; the tissues around the MBRs were homogeneous red-stained materials with no qualitative structure. High-power microscopy showed foam cells, suggesting a reaction after tumor treatment. Comparatively, many tumor cells were still present in the control and MBR groups. Immunohistochemistry (IHC) showed that the tumor cells in the control and MBR groups proliferated actively with a low rate of apoptosis, while the tumor cells in the MBR + AMF group were killed and transformed into an amorphous structure without nuclei, resulting in neither Ki67 nor TUNEL staining. Quantitative correlation analysis also showed the same results, with significant differences in the Ki67 index and apoptosis index between the MBR + AMF group and the control and MBR groups. These results indicated that MBRs can significantly inhibit the growth of bone tumors in load-bearing bone in situ, further illustrating the potential of MBRs in mutually-synergistic mild hyperthermia-starvation therapy for bone tumors in situ.

## Conclusion

In this study, injective phase-transform magnetic bone repair hydrogels (MBRs) were constructed by the rational integration of Fe_3_O_4_, MgCO_3_ particles and GOx-PLGA gels. The MBRs-based strategy was engineered to drive mutual synergistic hyperthermia**–**starvation therapy for OS treatment and augmented bone regeneration. In particular, the MBRs implants exhibited enhanced MTT effects activated by AMF exposure, which were superior to those of other OS treatments and had no deep tissue limitation. Subsequently, the released GOx effectively exhausted endogenous glucose to block the tumor cell energy supply and ATP generation. Notably, the current work has the following advantages. First, novel MBRs with the triple function of MTT and mutually synergistic starvation therapy were fabricated for anti-OS treatment. GOx-induced ATP supply restriction can also downregulate HSP expression, thereby reversing the heat tolerance of cancer cells and augmenting the MTT effect. The significant antitumor performance of the MBRs was confirmed by many systematic in vivo and in vitro experimental evaluations, which originated from the synergistic therapy of mild magnetic hyperthermia triggering glucose depletion, without obvious side effects. Second, once injected into the tumor tissue, MBRs with good fluidity rapidly underwent liquid solid transformation to fill irregular pathological bone defects and further contribute to the development of advanced biomaterial synthesis and preparation technologies for both OS elimination and bone rehabilitation. Third, the application of the as-fabricated MBRs was by in situ injection, which may also overcome limitations associated with surgical implants to facilitate a minimally invasive strategy. Moreover, the residual tumor model of bone tumors in situ on the tibial plateau more intuitively simulates the clinical pathophysiological process of bone tumors, further verifying that mutual synergistic hyperthermia**–**starvation therapy by MBRs could effectively kill residual tumors and inhibit tumor growth. In conclusion, triple-functional MBRs offer an advantageous candidate for the minimally invasive treatment of OS and offer an attractive prospect for further clinical translation.

## Experimental section

### Materials

PLGA (Mw: 40,000 Da, 50:50) was purchased from Jinan Daigang Biomaterial Co., China. NMP was purchased from Aladdin, China. Fe_3_O_4_ nanoparticles were obtained from Chengdu Aike Reagent, China. MgCO_3_ particles were obtained from Adamas, China. GOx was purchased from Sigma‒Aldrich (LA, USA). β-D-Glucose was obtained from Aladdin Reagent Co., Ltd. An alternating magnetic field (AMF) generator (frequency: 626 kHz, output current: 21.6 A, turns of coil: 2, coil length: 1 cm, coil diameter: 3 cm, field strength: 5.72 K/Am) was used as the irradiation source to drive mild hyperthermia.

### Preparation of PLGA and MBRs

In Scheme 1 A, the PLGA hydrogel was synthesized by adding PLGA to NMP at a conventional mass/volume ratio. As previously reported, PLGA was dissolved in NMP to obtain PLGA gels by mixing overnight in a shaking incubator at 37 °C. Then, a proportional amount of GOx was added to the PLGA gels to obtain GOx-PLGA gels. Fe3O4/GOx/MgCO3@PLGAliquid gels (MBRs) were synthesized by dispersing Fe_3_O_4_ and MgCO_3_ particles into the GOx-PLGA hydrogels at different mass ratios through mechanical stirring. As shown in Table 1, different liquid forms of MBR gels with good injection performance were prepared.

**Table 1:** Compositions of PLGA and MBR gels

Groups		Fe_3_O_4_ (wt%)	GOx (wt%)	MgCO_3_ (wt%)	PLGA (wt%)
PLGA		0	0	0	100
GOx-PLGA		0	1	0	99
MBRs	5%Fe_3_O_4_-MBRs	5	1	20	74
	10%Fe_3_O_4_-MBRs	10	1	20	69
	20%Fe_3_O_4_-MBRs	20	1	20	59

### Characterization

The general forms of the Fe_3_O_4_ nanoparticles, GOx and MgCO_3_ particles and the solid forms of the PLGA and MBR gels were recorded by digital images. To evaluate the stability, the photographic images of the MBRs at different time points after preparation were recorded. The microscopic views of all the above particles and the solid forms of the PLGA and MBR gels were chosen for structural and compositional analyses by scanning electron microscopy (SEM) (ZEISS 300, Germany) without and with AMF exposure. The elemental mapping analysis was obtained by energy dispersive spectroscopy (EDS) with the same parameters used for SEM. An automatic pore analyzer (AutoPore 9500, USA) was applied to acquire the pore structure data of the MBRs. A Fourier transform infrared (FTIR) spectrometer (Nicolet 6700) was used to acquire the infrared spectra. X-ray diffraction (XRD) experiments were conducted by Rigaku Ultima IV (Japan). X-ray photoelectron spectroscopy (XPS) was performed using a Thermo Scientific K-Alpha. Hysteresis loop analysis was conducted by Lakeshore 7404. The water contact angles (WCAs) of PLGA, GOx-PLGA, MgCO_3_@PLGA, Fe_3_O_4_@PLGA and MBRs were measured by an automatic contact angle meter (Dataphysics DCAT20, Germany). The thermal stability and practical amounts of the incorporated inorganic components of the MBRs were analyzed using thermogravimetric analysis (TGA) (TGA5500, USA). The iron concentrations of MBRs before and after AMF irradiation were quantitatively measured by inductively coupled plasma**–**optical emission spectrometer (ICP**–**OES).

### Cell culture and establishment of a 143B OS tumor-bearing nude mouse model

143B OS cells and multipotent immortalized mouse embryonic fibroblasts (iMEFs) were obtained from Chongqing Medical University (CQMU). α-Modified Eagle’s medium (α-MEM; Gibco, USA) supplemented with 100 U/mL penicillin (Beyotime, China), 0.1 mg/mL streptomycin (Beyotime, China) and 10% fetal bovine serum (FBS; Lonsera, Uruguay) was applied as culture medium. 143B cells and iMEFs were cultured in culture medium at 37 °C with 5% carbon dioxide (CO_2_). For the osteogenic differentiation study, osteogenic induction medium was prepared from the abovementioned culture medium by adding 50 µmol/L vitamin C (Sigma), 10 mmol/L β-sodium glycerophosphate (Solarbio, China), and 10 nmol/L dexamethasone (Sigma).

SPF BALB/c-nude mice (nude mice) (4–6 weeks old, 14–16 g) were purchased from Chongqing Ensiweier Biotechnology Co., Ltd. (Chongqing, China) and fed at the Animal Experiment Center of CQMU. All mice were conventionally raised for one week. 143B human OS cells (1 × 10^6^ cells) were subcutaneously injected into the backs of mice to establish animal tumor xenografts. When the tumor volume reached approximately 100 mm^3^, the mice were used for further experiments. All animal studies were performed following protocols approved by the Animal Ethics Committee of CQMU and Chongqing Traditional Chinese Medicine Hospital.

### In vitro and in vivo mild magnetothermal performance of various forms of MBRs

To evaluate the mild magnetothermal performance of the MBRs, varying mass ratios of Fe_3_O_4_ nanoparticles (5%, 10% and 20%) and different volumes of MBRs (50 µL, 75 µL, or 100 µL) were placed into ex vivo bovine liver and saline solution. After treatment, the ablation areas were recorded. The 75 µL MBR gel was injected into the tumor. After a day of full solidification of the materials, the 143B osteosarcoma tumor-bearing nude mice were placed into the center of an electromagnetic induction heating coil for mild magnetic-induced hyperthermia. To record the magnetic thermal performance of MBRs exposed to the AMF, a far-infrared thermometer (FOTRIC225, ZXF Laboratories, US) was used, and the temperature changes were analyzed using thermal images (AnalyzIR 7.1 software). The above experiments were performed three times in each group. The magnetic thermal-triggered GOx release behavior was evaluated by a BCA protein assay with or without magnetic hyperthermia (MH).

### Evaluation of mild hyperthermia-triggered GOx release to induce starvation therapy

#### Mild hyperthermia-triggered GOx release

To evaluate the GOx release behavior, the prepared 200 µL MBRs gel was placed in a 15 mL centrifuge tube with 10 mL PBS solution. After being exposed to AMF for different cycles, the mixture was shaken at 200 rpm, 37 ℃. At predetermined time points, the supernatant was taken for GOx concentration determination using the BCA kit.

#### Consumption and generation of O_2_in vitro

The oxygen concentration was evaluated via a portable dissolved oxygen meter (550 A, YSI, Ohio, USA) in several groups: H_2_O_2_, MBRs + H_2_O_2_, MBRs, MBRs + H_2_O_2_ + AMF, glucose, GOx + glucose, MBRs + GOx + glucose, and MBRs + GOx + glucose + AMF. The concentrations of GOx solution, glucose and H_2_O_2_ in Fig. 3 were 0.3 mg/mL, 10 mg/mL and 5 µmol/mL, respectively. To maintain the reaction temperature at 37 °C or 40–45 °C (AMF groups), all experiments were performed in a water bath or exposed to an AMF under certain conditions.

#### Generation of H_2_O_2_ and gluconic acid in vitro

In this part, the ability of GOx to transform glucose into H_2_O_2_ and gluconic acid and the mild hyperthermia-triggered GOx release to induce catalytic capability for the disproportionation of H_2_O_2_ were confirmed. The H_2_O_2_ content was gauged by the H_2_O_2_ assay kit according to the manufacturer’s instructions. In terms of the GOx-mediated catalytic reaction, the acidity in the microenvironment was increased by the generation of gluconic acid, as shown by the reduction in the pH value, which was utilized for gluconic acid monitoring. The experiments were implemented in a water bath or exposed to an AMF to keep the reaction temperature at 37 °C or 40–45 °C (AMF groups) according to the purpose of the experiment.

#### Generation of ATP and expression of HSP70 protein in vitro

An ATP assay kit (Beyotime, China) was used to measure the intracellular ATP level. Briefly, 143B cells (2 × 10^5^ per well) were seeded into 6-well plates and incubated with fresh medium with different treatment groups of control (PBS), Fe_3_O_4_/MgCO_3_@PLGA, and MBRs for 12 and 24 h. Then, the ATP level was measured according to the manufacturers’ instructions. For the analysis of the expression level of HSP70, 143B cells were treated with normal medium (control), Fe_3_O_4_/MgCO_3_@PLGA, Fe_3_O_4_/MgCO_3_@PLGA + AMF, MBRs, or MBRs + AMF, and the HSP levels of different groups were determined by Western blotting.

### In vitro mutual synergistic therapeutic efficacy of MBRs

The cytotoxicity of MBRs was evaluated by flow cytometry analysis. 143B cells (2 × 10^5^ per well) were cultured in 6-well plates. To evaluate the cytotoxicity of the MBRs, culture medium with different treatment groups of control (PBS), AMF, GOx, Fe_3_O_4_/MgCO_3_@PLGA, and MBRs were added and incubated for 24 and 48 h. Afterward, the cells were collected and stained with Annexin V-FITC and PI for 15 min in the dark. The mutual synergistic therapeutic efficacy of the MBRs was evaluated by the standard CCK-8 assay and flow cytometry analysis. 143B cells were added to Fe_3_O_4_/MgCO_3_@PLGA (FM@P) and MBRs for 24 and 48 h. Additionally, the cells were exposed to an AMF via multicycle magnetic hyperthermia therapy. To further assess the therapeutic effect of five AMF cycles, 143B cells (1 × 10^4^ per plate) were cultured in 35 mm glass-bottom dishes overnight. After various treatments, the cells were rinsed with PBS and incubated with calcein-AM/PI staining solution (Beyotime, China) for 30 min. Subsequently, the cells were visualized by a fluorescent cell imager.

### In vivo mutual synergistic therapeutic efficacy and biosafety of MBRs

After the tumor volume reached approximately 100 mm^3^, the mice were divided randomly into four groups (n = 5): (1) the tumor group (as a control); (2) the MBR group; (3) the Fe_3_O_4_/MgCO_3_@PLGA + AMF group; and (4) the MBR + AMF group. The mice received intratumor injections of 75 µL of different materials, including PBS, MBRs or Fe_3_O_4_/MgCO_3_@PLGA. A day after the intratumor injection and after the materials turned solid, an AMF was applied to irradiate the tumors for 5 cycles. The tumor-site temperature elevation was recorded by a thermal infrared imaging camera. Moreover, the mouse body weights and morbidity-free survival were observed during 14 days of follow-up, as shown in Fig. 5A. To further evaluate the therapeutic efficacy in vivo, the tumors were subjected to hematoxylin-eosin (HE) staining at 24 h posttreatment. In addition, TdT-mediated dUTP nick-end labeling (TUNEL), KI67, HSP70 and HSP90 assays were carried out to quantitatively evaluate the apoptosis/proliferation and expression of HSP proteins in the tumors. Moreover, the major organ (heart, liver, spleen, lung, kidneys) and serum samples, including aspartate transaminase (AST), alanine aminotransferase (ALT), creatinine (CR), blood urea nitrogen (BUN), ang creatine kinase (CK), were collected, and the biochemical and routine blood tests were analyzed using a biochemical autoanalyzer to further assess the biosafety of the applied materials.

### Evaluation of bone regeneration by MBRs

#### In vitro osteogenic differentiation

Multipotent immortalized mouse embryonic fibroblasts (iMEFs) (1 × 10^4^ per well) were seeded into 24-well plates and cultured overnight. To evaluate the osteogenic property of the MBRs, osteogenic induction medium with different treatment groups, namely, control (PBS), PLGA, Fe_3_O_4_/MgCO_3_@PLGA and MBRs were added and incubated for 7 days and 14 days. Alkaline phosphatase (ALP) activity was measured using an ALP assay kit (Beyotime, China). For ALP staining, the cells were stained with a BCIP/NBT ALP color development kit (Beyotime, China) after fixation with 4% paraformaldehyde for 15 min. For alizarin red S (ARS) staining, cells were fixed as previously mentioned and stained with alizarin red working solution (Beyotime, China) according to the manufacturer’s instructions. For quantification, the stained nodules were dissolved in 10% acetic acid, and the absorbance was detected at 405 nm.

#### Critical-sized calvarial defect model

A skull defect rat model was exploited to elucidate the osteogenic peculiarity of the MBRs. Rat bilateral critical-sized cranial defects 5 mm in diameter were established on two sides of the rats’ skulls with a trephine bur. The model rats were randomly divided into three groups and treated as follows: (1) the self-control group: the defects were left unfilled; (2) the PLGA group: the right defect was injected with 30 µL of PLGA gels; and (3) the MBR group: the right defect was injected with the 30 µL of MBRs. After the MBRs were phase-transformed by dipping in saline for 3–5 min, the surgical sites were layer-sutured.

#### In vivo bone regeneration

Three rats per group were sacrificed at four, eight and twelve weeks postsurgery, and calvarial bone defect samples were collected and fixed in 4% paraformaldehyde for further analysis. A micro-CT system (µCT100, Scanco Medical, Switzerland) was used to scan the samples, and quantitative analysis and 3D reconstruction of bone regeneration were carried out. Scoring guides were referenced to evaluate the type of bone defect repair according to the micro-CT images. In addition, ImageJ software (V1.52, NIH, USA) was used to analyze the bone formation and coverage percentage. After immersion in ethylenediaminetetraacetic acid (EDTA; 0.5 M) solution for several weeks, decalcified samples were prepared for subsequent histological examination. H&E, safranin O-fast green and Masson trichrome staining were performed.

### Evaluation of the anti-residual bone tumor effect of MBRs in situ

#### Establishment of an in situ residual bone tumor model in the rabbit tibial plateau

New Zealand rabbits that were 2 months old and weighed 2.0-2.5 kg were used in this experiment. Tumor tissues were removed from the VX2 tumor-bearing rabbits, and the tumor masses were cut and separated into pieces approximately 1 mm^3^ in size. Before anesthesia, all experimental rabbits were fasted from solids and liquids. After the experimental rabbits were anesthetized, tumor tissue was inserted into the tibial plateau of the rabbits through a coaxial puncture needle, and the intraosseous needle passage was sealed with a gelatin sponge. On the 14th day after implantation, the tumor size was measured by CT scanning. When the bone destruction volume of the tibial plateau reached approximately 150 mm^3^, surgical resection was performed, starting with a one centimeter longitudinal incision on the medial side of the proximal tibia. Then, the skin was cut to expose the deep muscles, the middle portion of the muscles along the bone surface was dissected, and the cortical bone of the medial tibia was exposed using a grinding drill and a small bone knife. Then, the cancellous bone of the tibial plateau and the tumor tissue within were exposed, and the visible tumor tissue was removed to establish a residual bone tumor. Penicillin was used for three days after surgery to prevent infection.

#### In vivo mutual synergistic therapeutic efficacy

The rabbits in which the in situ residual bone tumor model was established were randomly divided into three groups (n = 5), namely, the control group, the MBR group, and the MBR + AMF group. Animals in each group received different treatments, that is, no intervention, filling only with MBRs, and exposure to AMF after filling with MBRs. In the MBR + AMF group, the temperature elevation at the tumor site in the rabbit leg was recorded by a thermal infrared imaging camera. Before and 3 weeks after treatment, CT scans of the tibia of the experimental rabbits in the different treatment groups were performed to observe the growth of the residual tumors, and 3D reconstruction was used to measure the bone destruction more clearly. At 3 weeks after treatment, the bone tissue around the tibial plateau in the rabbits was collected for H&E staining to evaluate the therapeutic efficacy. Moreover, TUNEL and Ki-67 assays were performed to evaluate the proliferation and apoptosis of tumor cells.

### Statistical analysis

All data were analyzed by GraphPad Prism 8.0 software. Values are shown as the means ± standard deviations (SDs). An independent-samples t test and one-way ANOVA were used for intergroup comparisons. Statistical significance: *p < 0.05, ** *p* < 0.01, *** *p* < 0.001, and # *p* < 0.05, ## *p* < 0.01, ### *p* < 0.001.

## Electronic supplementary material

Below is the link to the electronic supplementary material.

Supplementary Material 1
