# Supplementary material for: Engineering a triple-functional magnetic gel driving mutually-synergistic mild hyperthermia-starvation therapy for osteosarcoma treatment and augmented bone regeneration
Source: J Nanobiotechnology. 2023 Jun 26;21:201. doi: 10.1186/s12951-023-01955-7 (PMC10291780; doi:10.1186/s12951-023-01955-7)
Supplement: Supplementary file 1 — Supplementary Material 1 [file 12951_2023_1955_MOESM1_ESM.docx]

***Supplementary data***

**Engineering a Triple-Functional Magnetic Gel Driving Mutually-synergistic Mild Hyperthermia-Starvation Therapy for Osteosarcoma Treatment and Augmented Bone Regeneration**

Kexiao Yu ^a,^*, Hang Zhou ^b,e,^*, Yamei Xu ^c^, Youde Cao ^c^, Yuanyi Zheng ^d,#^ and Bing Liang ^c,e,#^

a Department of Orthopedics, Chongqing Traditional Chinese Medicine Hospital, No. 6 Panxi Seventh Branch Road, Jiangbei District, Chongqing 400021, P. R. China.

b Department of Orthopedics, Second Affiliated Hospital of Chongqing Medical University, 76 Linjiang Road, Yuzhong Distinct, Chongqing, 400010, P. R. China.

c Department of Pathology, College of Basic Medicine, Molecular Medicine Diagnostic and Testing Center, Chongqing Medical University, 1 Yixueyuan Road, Yuzhong Distinct, Chongqing, 400016, P.R. China.

d Shanghai Institute of Ultrasound in Medicine, Department of Ultrasound in Medicine, Shanghai Jiao Tong University Affiliated Sixth People’s Hospital, 600 Yishan Road, Xuhui Distinct, Shanghai, 200233, P. R. China.

e State Key Laboratory of Ultrasound in Medicine and Engineering, Institute of Ultrasound Imaging, Chongqing Medical University, Chongqing, 400010, People's Republic of China.

* Kexiao Yu and Hang Zhou are co-first authors who contributed equally to this study.

# Corresponding authors: Dr. Bing Liang, Department of Pathology, College of Basic Medicine, Chongqing Medical University, 1 Yixueyuan Road, Yuzhong Distinct, Chongqing, 400016, P.R. China. E-mail: doctorliang51@163.com; Prof.Yuanyi Zheng, Shanghai Institute of Ultrasound in Medicine, Shanghai Jiao Tong University Affiliated Sixth People’s Hospital, 600 Yishan Road, Xuhui Distinct, Shanghai, 200233, P. R. China. E-mail: [zhengyuanyi@163.com](mailto:zhengyuanyi@163.com).

***Supplementary data***


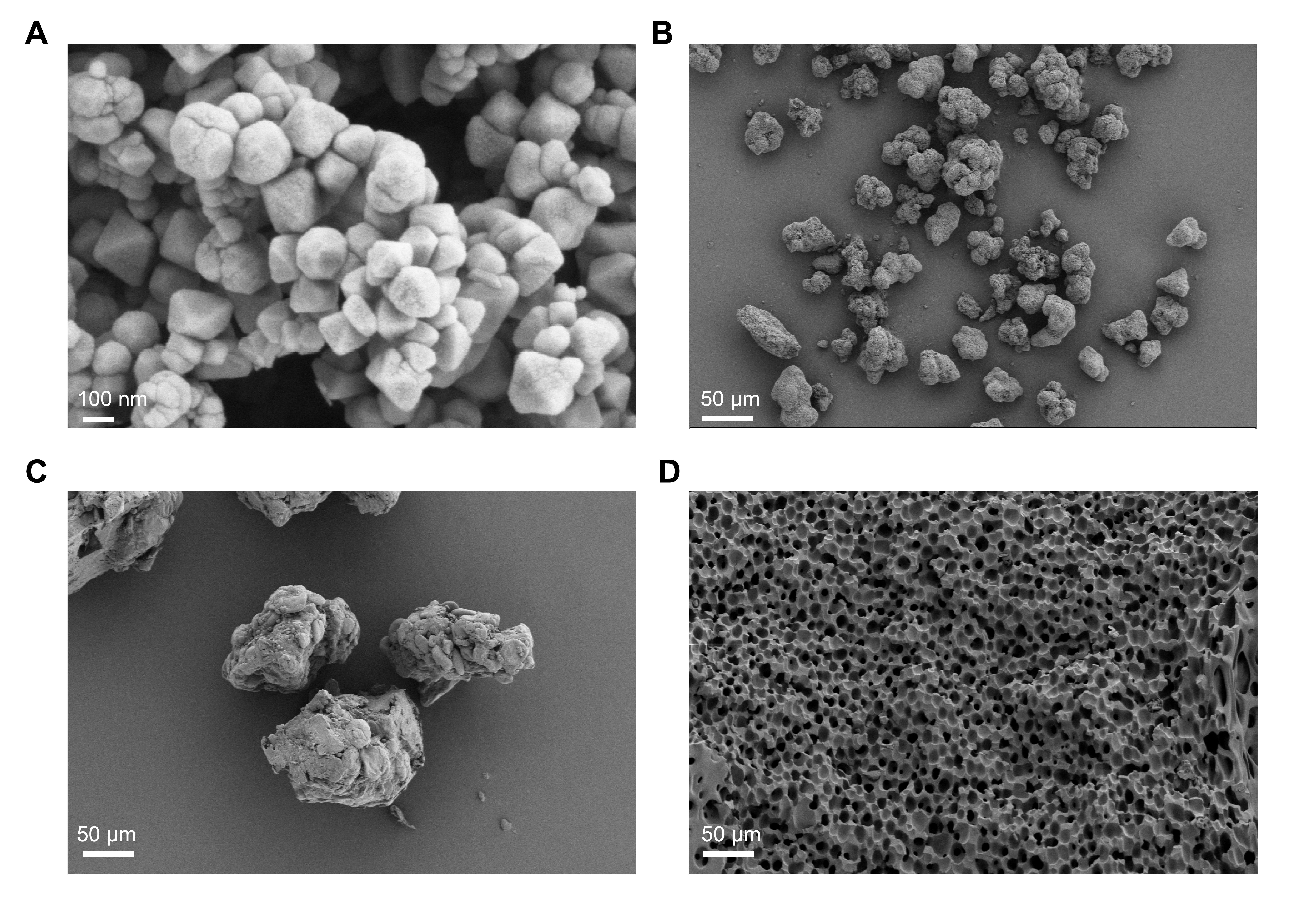


**Figure S1.** SEM images of (A) Fe_3_O_4_ nanoparticles, (B) GOx particles, (C) MgCO_3_, (D) solid form of PLGA.


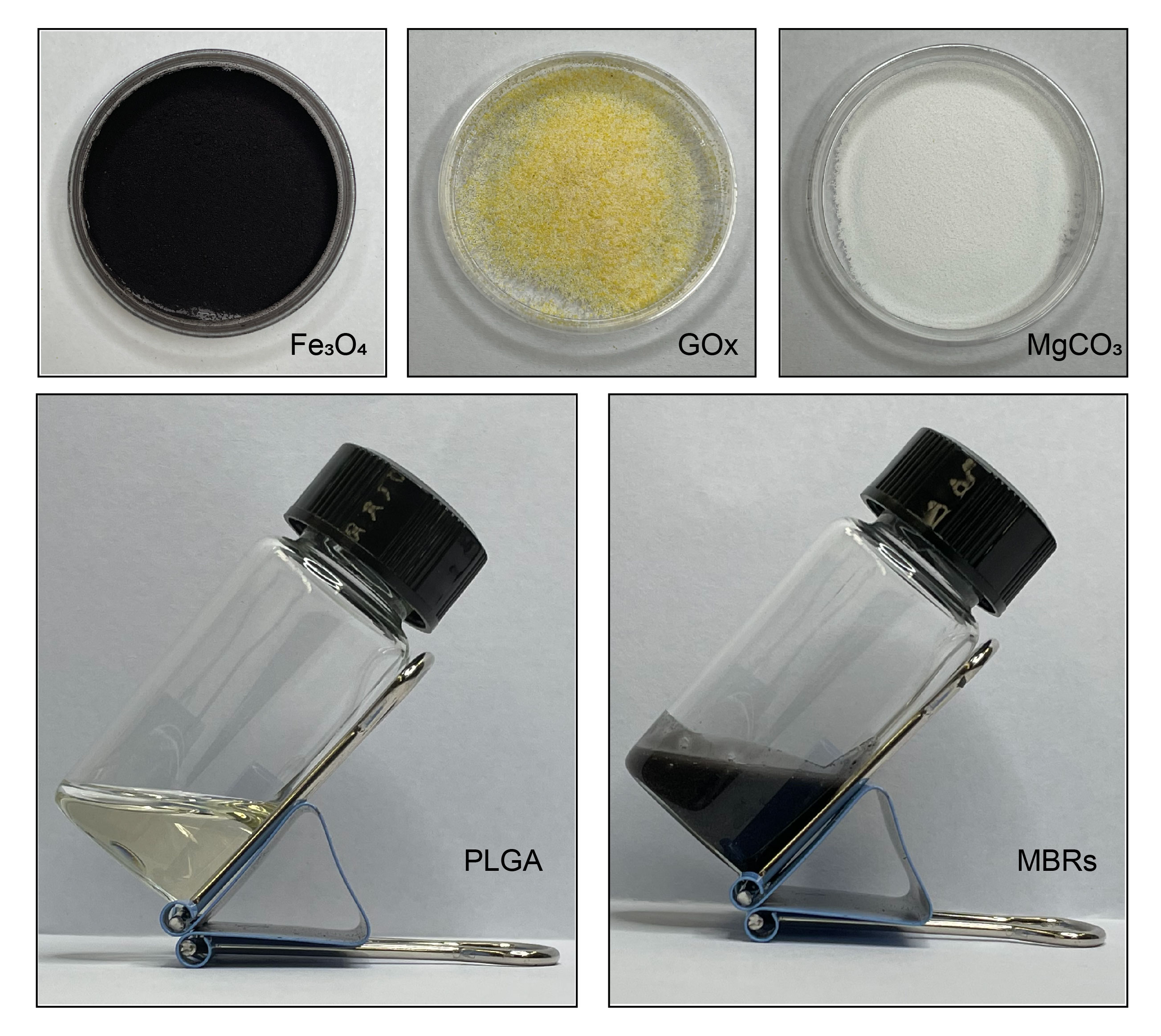


**Figure S2.** Digital images of Fe_3_O_4_, GOx, MgCO_3_, PLGA gels and MBR gels.


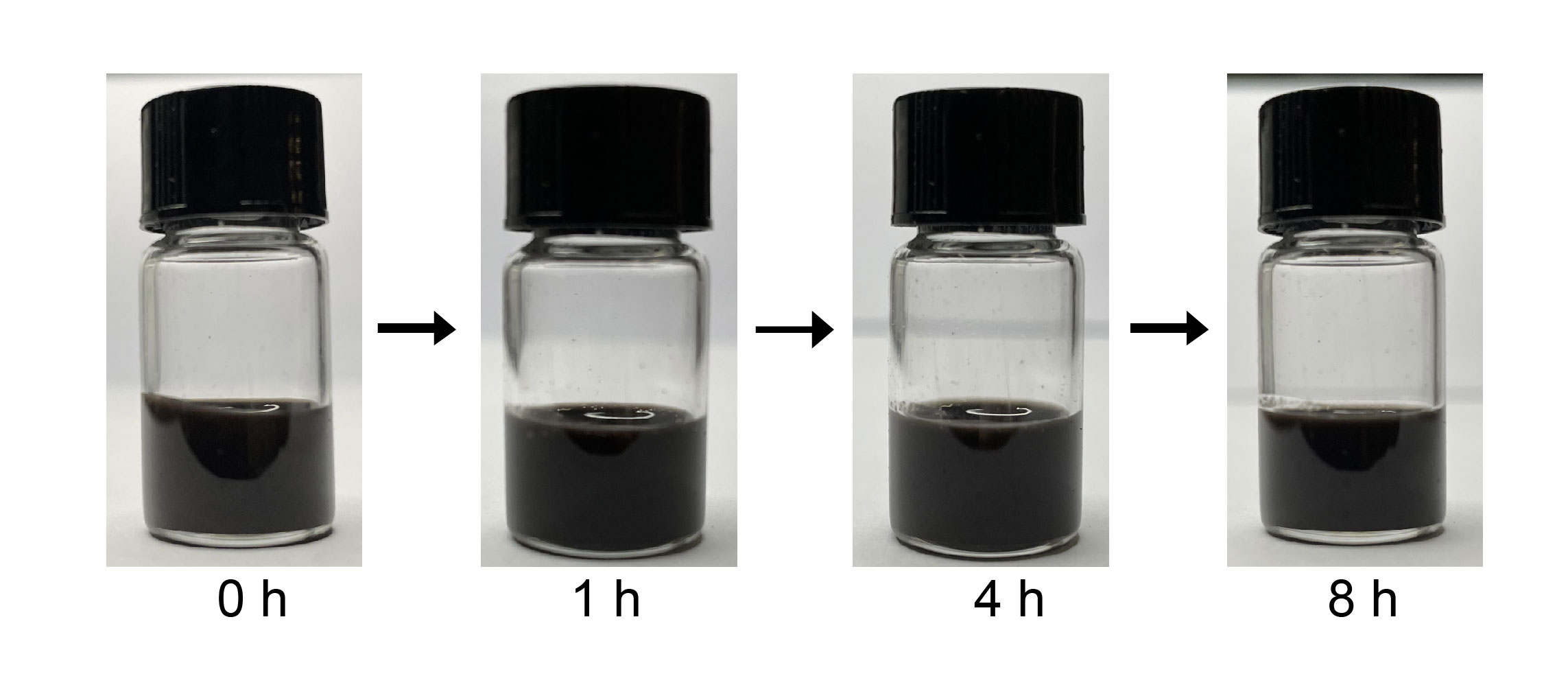


**Figure S3**. Digital images of liquid MBRs with different time periods.


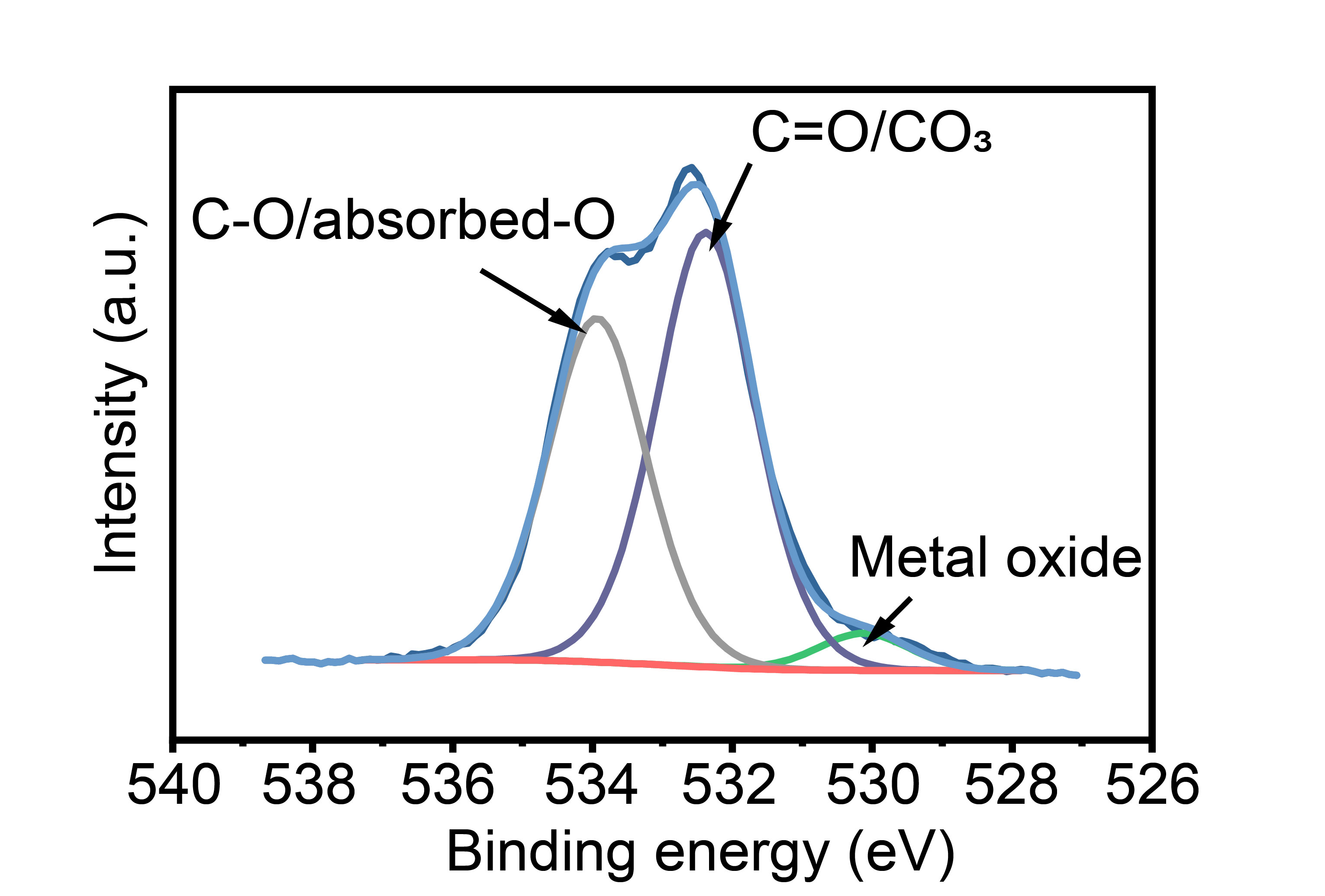


**Figure S4.** XPS spectra of metal oxide.


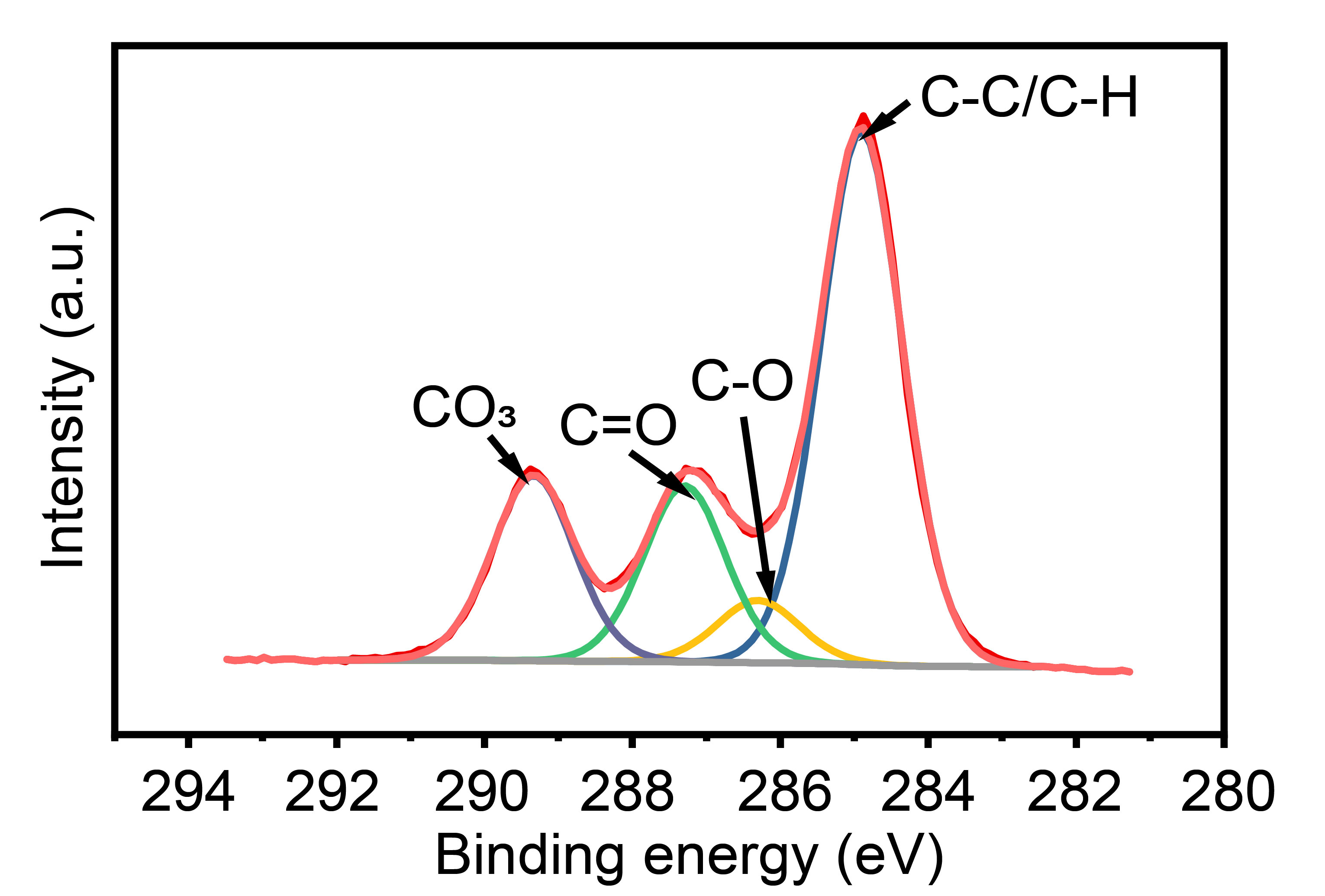


**Figure S5.** XPS spectra of carbonate.


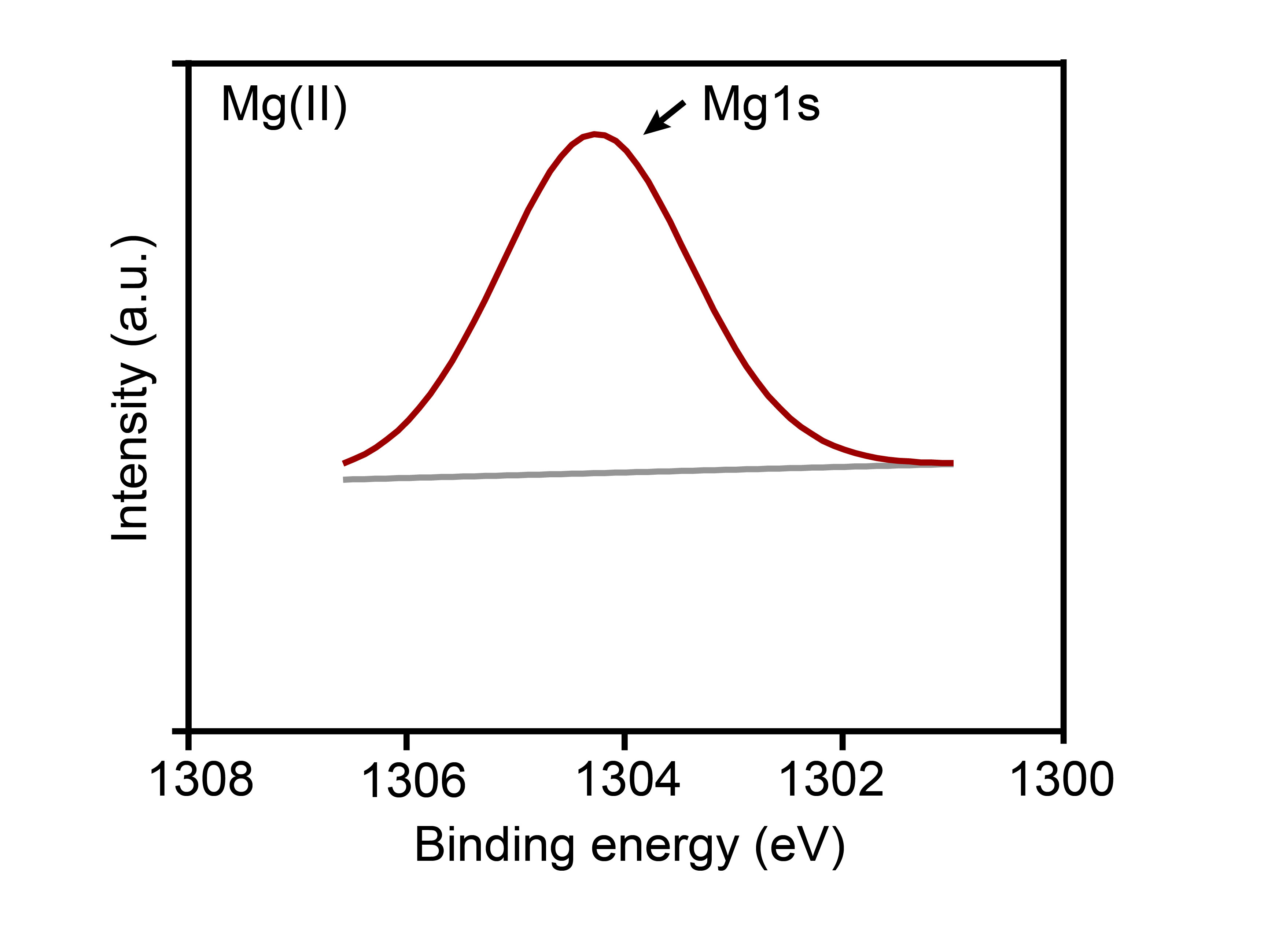


**Figure S6.** Mg 1s XPS of MBRs.


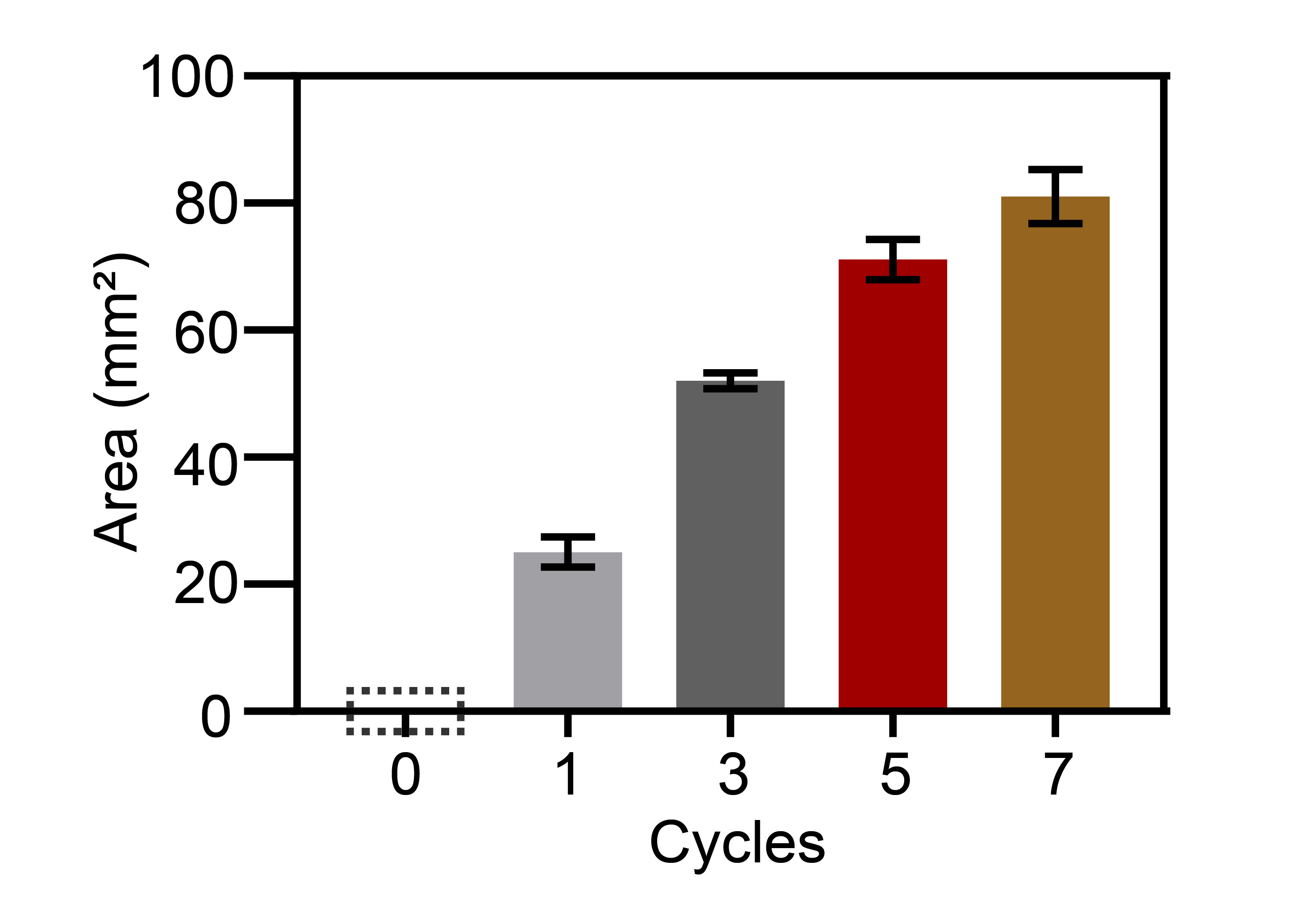


**Figure S7.** The ablation areas of excised bovine liver containing 75 μL 10% Fe_3_O_4_-MBRs for “on-off” model magneto-thermal treatment of several cycles.


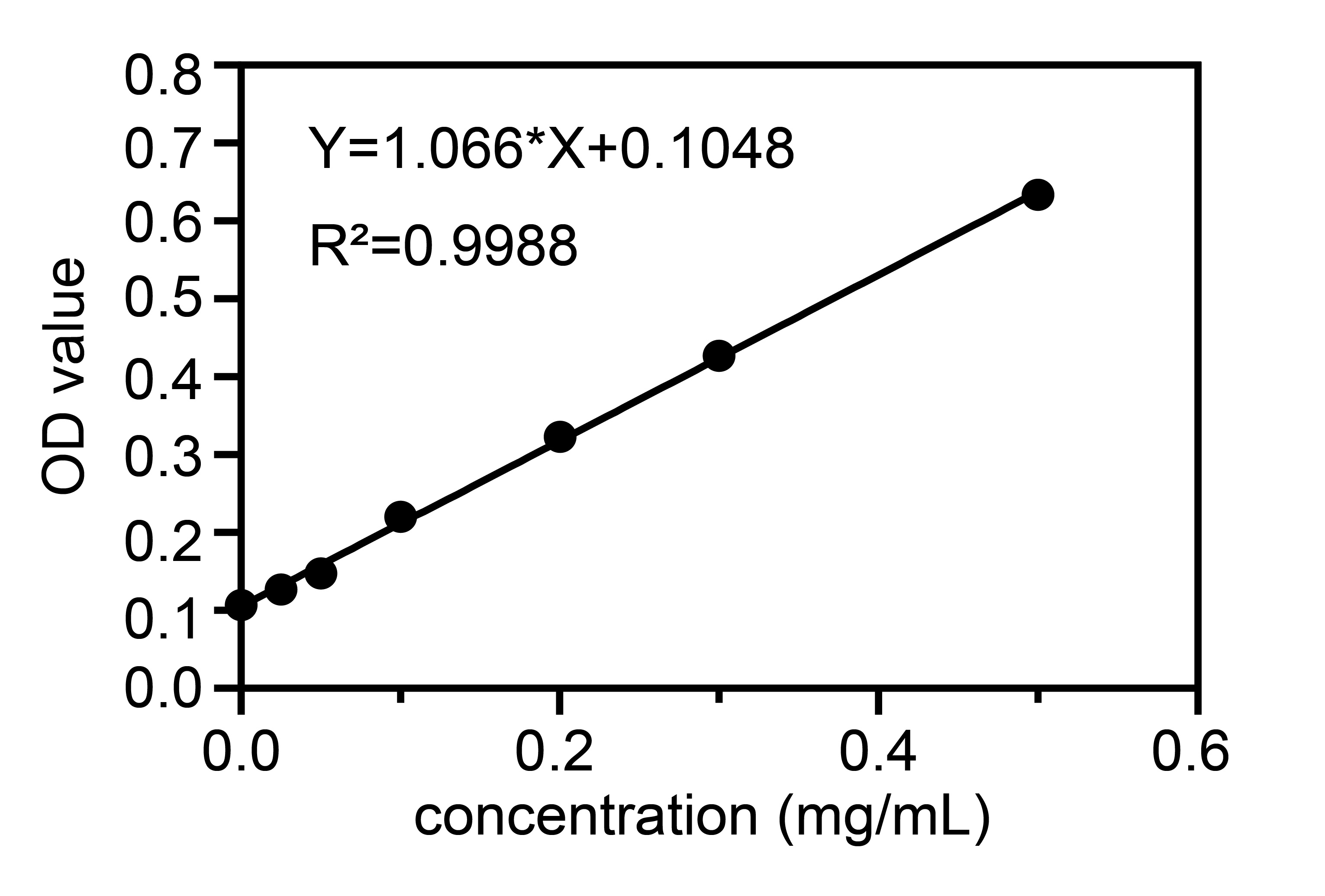


**Figure S8.** The standard curve of GOx solution.


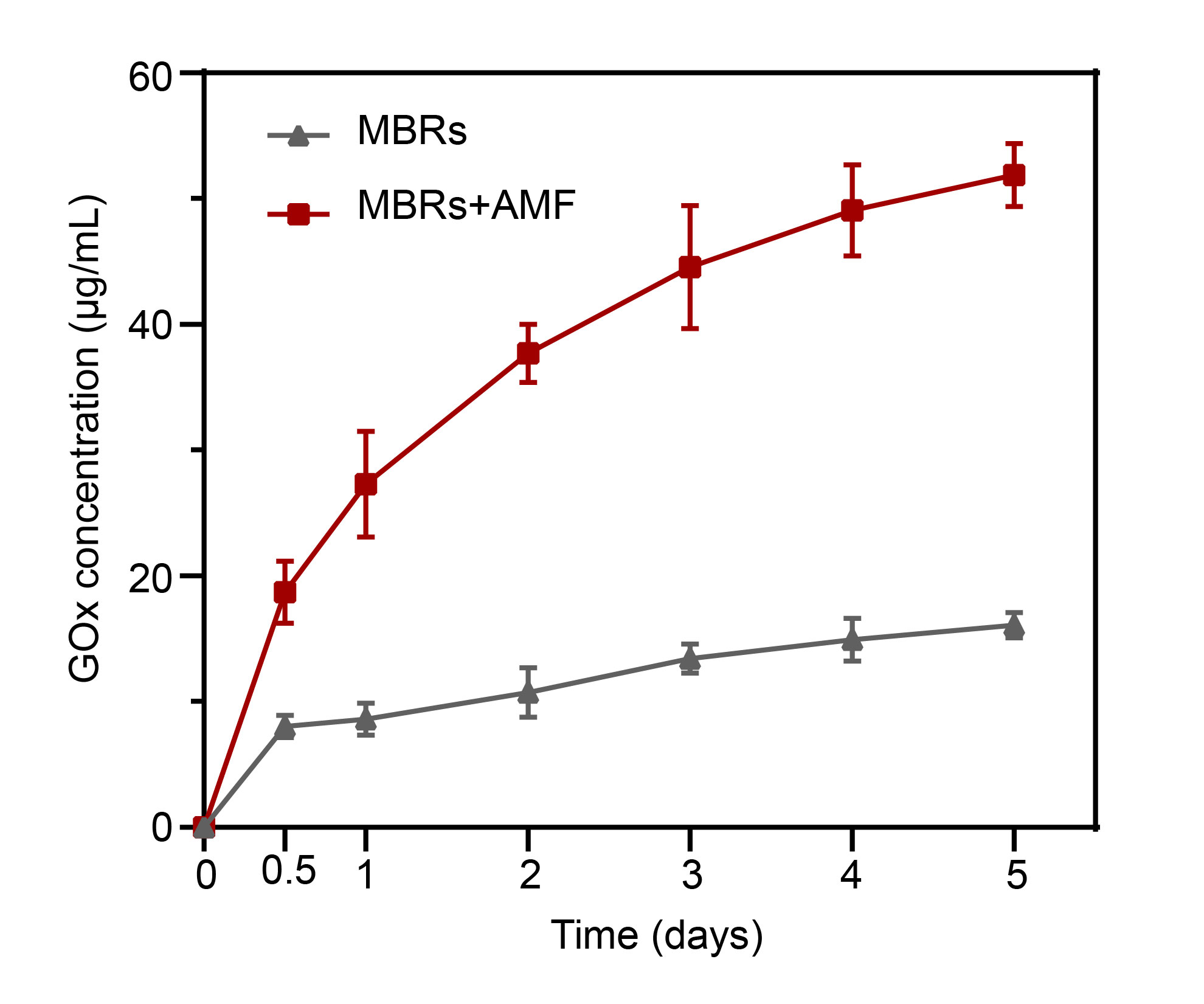


**Figure S9**. The GOx release behavior of MBRs gel with or without AMF.


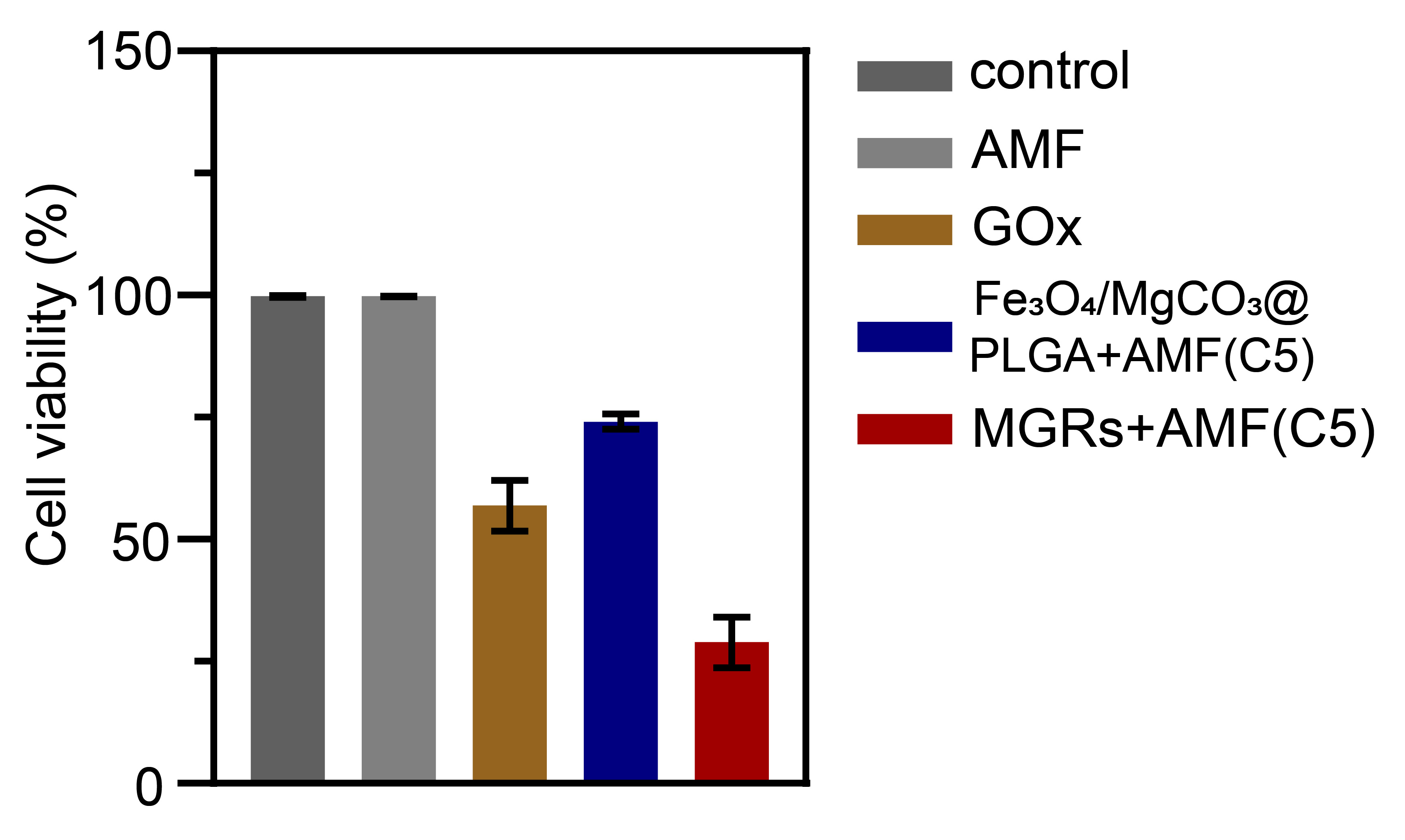


**Figure S10.** The corresponding quantitative analyses of Calcein-AM (green)/PI (red) staining images of 143B cells after various treatments based of Figure 4E.


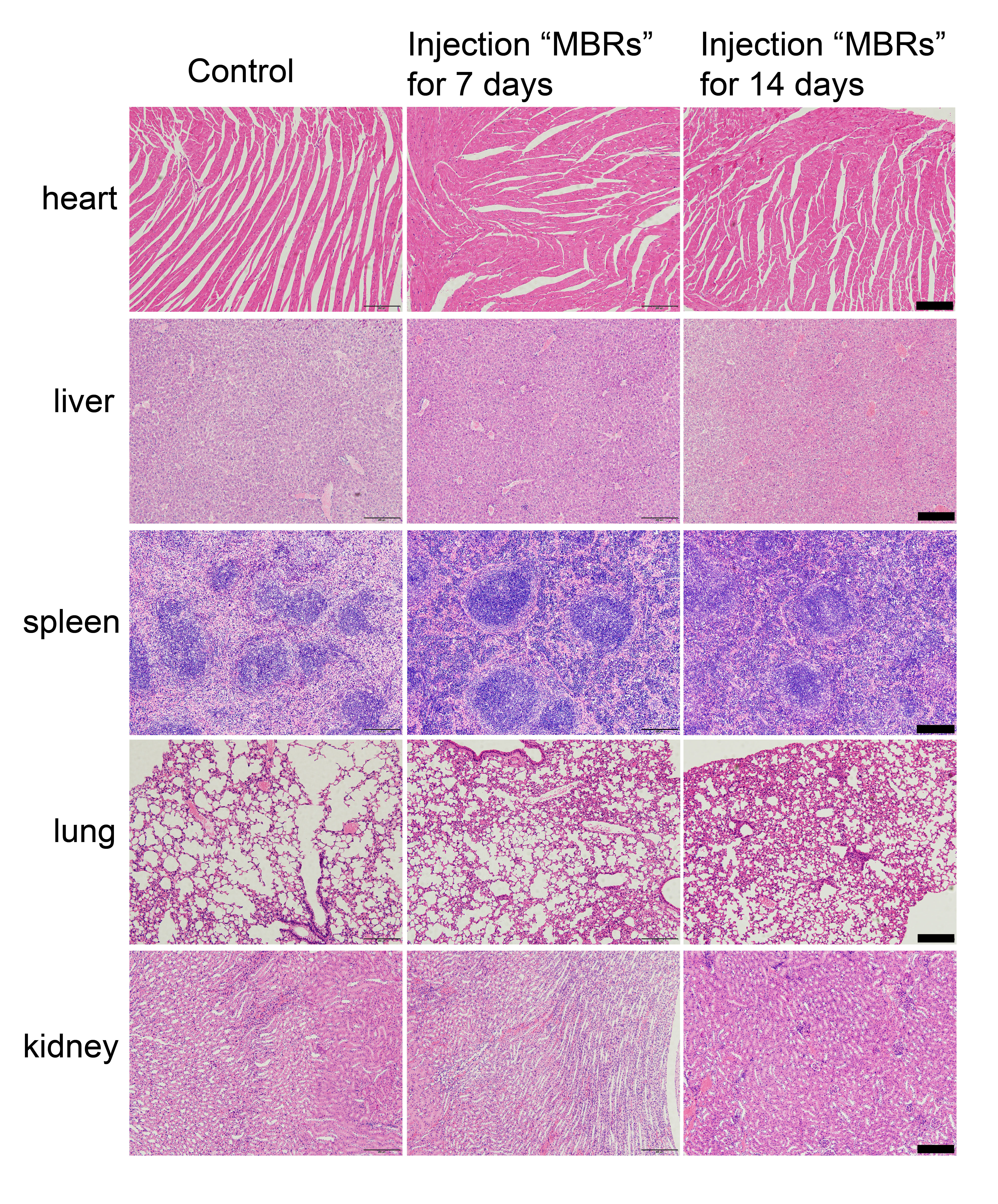


**Figure S11.** H&E staining images of the major organs of mice after i.t. administration of 75 μL MBRs for several time points of feeding (Scale bars: 50 μm).


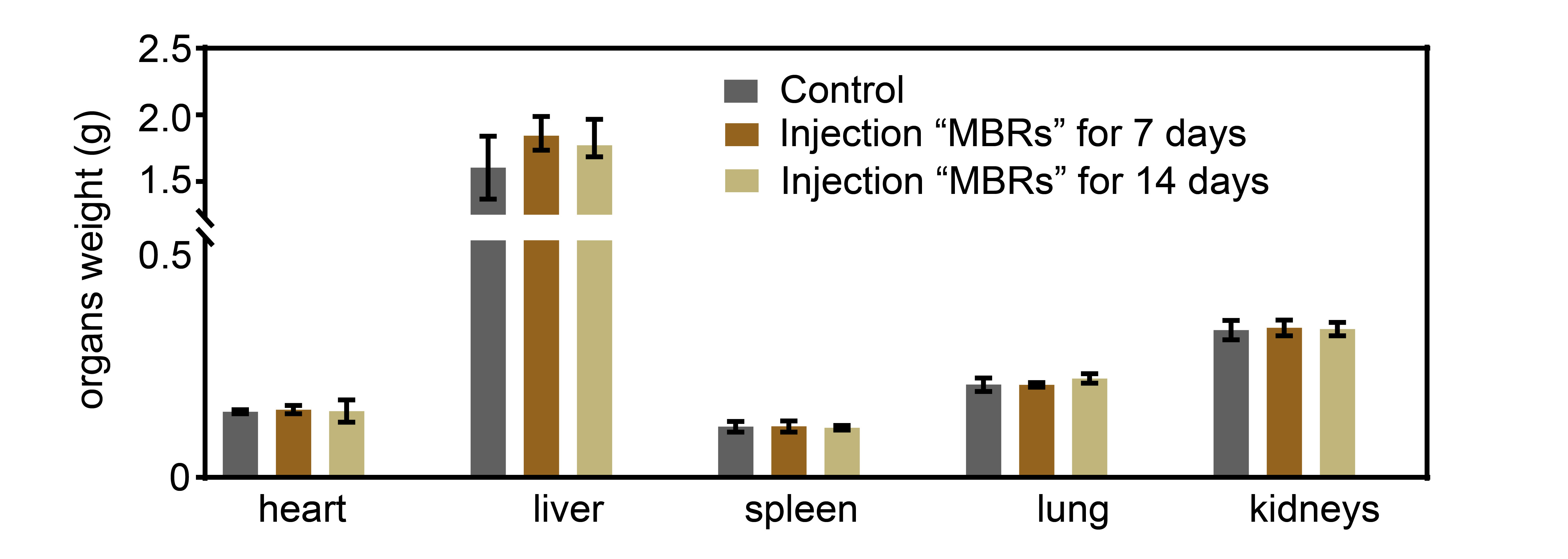


**Figure S12.** The corresponding organ weighs.


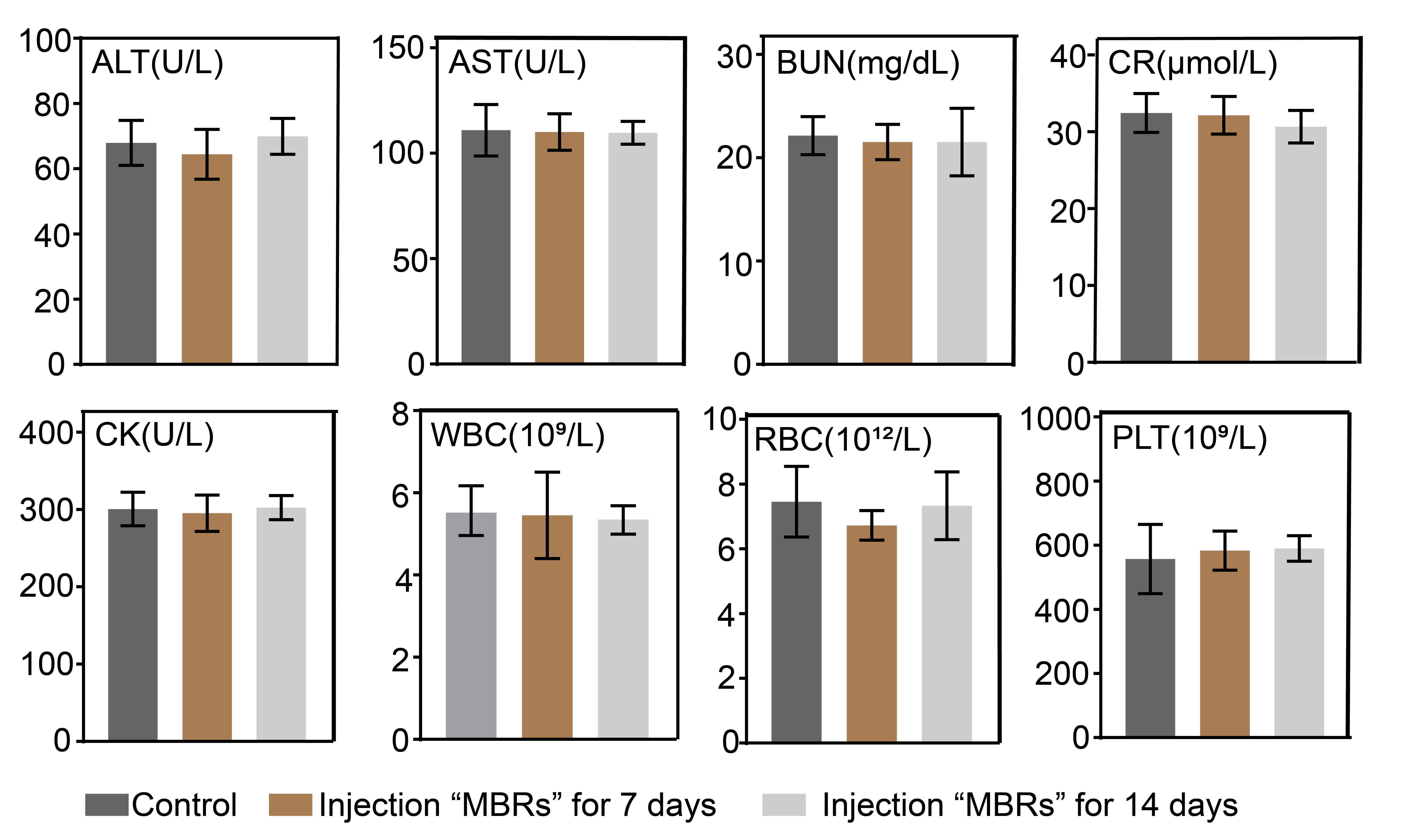


**Figure S13.** Hematological and blood biochemical test of mice after i.t. administration of MBRs at different time intervals.

**
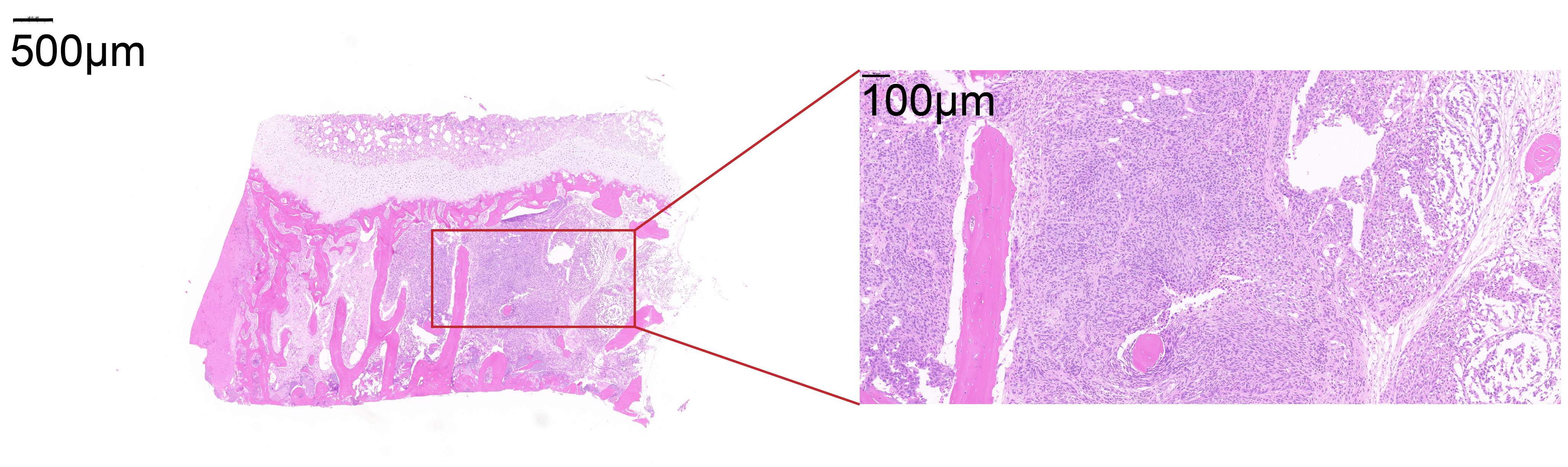
**

**Figure S14.** H&E staining images of tibial plateau bone tissue with tumor (Scale bars: 500 μm and 100 μm).
